# Development of Predictive Models to Inform a Novel Risk Categorization Framework for Antibiotic Resistance in *Escherichia coli–*Caused Uncomplicated Urinary Tract Infection

**DOI:** 10.1093/cid/ciae171

**Published:** 2024-04-04

**Authors:** Ryan K Shields, Wendy Y Cheng, Kalé Kponee-Shovein, Daniel Indacochea, Chi Gao, Fernando Kuwer, Ashish V Joshi, Fanny S Mitrani-Gold, Patrick Schwab, Diogo Ferrinho, Malena Mahendran, Lisa Pinheiro, Jimmy Royer, Madison T Preib, Jennifer Han, Richard Colgan

**Affiliations:** Department of Medicine, University of Pittsburgh, Pittsburgh, Pennsylvania, USA; Analysis Group, Inc., Boston, Massachusetts, USA; Analysis Group, Inc., Boston, Massachusetts, USA; Analysis Group, Inc., Boston, Massachusetts, USA; Analysis Group, Inc., Boston, Massachusetts, USA; Analysis Group, Inc., Boston, Massachusetts, USA; GSK, Collegeville, Pennsylvania, USA; GSK, Collegeville, Pennsylvania, USA; GSK, Collegeville, Pennsylvania, USA; GSK, Collegeville, Pennsylvania, USA; Analysis Group, Inc., Boston, Massachusetts, USA; Analysis Group, Inc., Boston, Massachusetts, USA; Analysis Group, Inc., Boston, Massachusetts, USA; GSK, Collegeville, Pennsylvania, USA; GSK, Collegeville, Pennsylvania, USA; Department of Family Medicine, University of Maryland School of Medicine, Baltimore, Maryland, USA

**Keywords:** uncomplicated urinary tract infection, antibiotic resistance, predictive modeling, trimethoprim-sulfamethoxazole, fluoroquinolone

## Abstract

**Background:**

In clinical practice, challenges in identifying patients with uncomplicated urinary tract infections (uUTIs) at risk of antibiotic nonsusceptibility may lead to inappropriate prescribing and contribute to antibiotic resistance. We developed predictive models to quantify risk of nonsusceptibility to 4 commonly prescribed antibiotic classes for uUTI, identify predictors of nonsusceptibility to each class, and construct a corresponding risk categorization framework for nonsusceptibility.

**Methods:**

Eligible females aged ≥12 years with *Escherichia coli–*caused uUTI were identified from Optum's de-identified Electronic Health Record dataset (1 October 2015–29 February 2020). Four predictive models were developed to predict nonsusceptibility to each antibiotic class and a risk categorization framework was developed to classify patients' isolates as low, moderate, and high risk of nonsusceptibility to each antibiotic class.

**Results:**

Predictive models were developed among 87 487 patients. Key predictors of having a nonsusceptible isolate to ≥3 antibiotic classes included number of previous UTI episodes, prior β-lactam nonsusceptibility, prior fluoroquinolone treatment, Census Bureau region, and race. The risk categorization framework classified 8.1%, 14.4%, 17.4%, and 6.3% of patients as having isolates at high risk of nonsusceptibility to nitrofurantoin, trimethoprim-sulfamethoxazole, β-lactams, and fluoroquinolones, respectively. Across classes, the proportion of patients categorized as having high-risk isolates was 3- to 12-fold higher among patients with nonsusceptible isolates versus susceptible isolates.

**Conclusions:**

Our predictive models highlight factors that increase risk of nonsusceptibility to antibiotics for uUTIs, while the risk categorization framework contextualizes risk of nonsusceptibility to these treatments. Our findings provide valuable insight to clinicians treating uUTIs and may help inform empiric prescribing in this population.

## Teaser

We demonstrate how predictive modeling can mitigate inappropriate prescribing in uncomplicated urinary tract infection by providing insight into factors that increase risk of nonsusceptibility to commonly prescribed antibiotics and developing a risk categorization framework that may improve empiric treatment strategies.

Urinary tract infections (UTIs) are the most common outpatient infections in the United States (US), with an estimated lifetime incidence of 50%‒60% in female adults [1]. Approximately 80% of UTIs are classified as uncomplicated UTI (uUTI), which is one of the most common indications leading to antibiotic prescriptions in females [2, 3]. The Infectious Diseases Society of America (IDSA) guidelines recommend nitrofurantoin (NTF), trimethoprim-sulfamethoxazole (SXT), and fosfomycin as first-line treatments, while antibiotics such as β-lactams and fluoroquinolones are recommended as alternative options [2, 3]. Despite guideline recommendations, prescribing practices not aligned with IDSA guidelines are common [4], with 86.1% of patients prescribed alternative antibiotic agents as first-line therapy [4, 5]. These inappropriate prescribing practices contribute to increased healthcare costs and development of antimicrobial resistance (AMR), which leads to treatment failure, persistent uUTI symptoms, and adverse events [4, 6–10].

Approximately 80% of uUTIs are caused by *Escherichia coli* (*E. coli*), and rising prevalence of AMR is a growing concern [11, 12]. A recent US study of urinary *E. coli* isolates from female outpatients ≥12 years of age found that 25.4%, 21.1%, and 3.8% were nonsusceptible to SXT, fluoroquinolones, and NTF, respectively, and 6.4% had extended-spectrum β-lactamase production [13]. In clinical practice, challenges in identifying patients with uUTI at risk of having a nonsusceptible isolate to commonly prescribed antibiotics can lead to inappropriate empiric prescribing. One novel method to help inform empiric prescribing by clinicians in uUTI is using a data-driven approach, such as predictive modeling, to identify patients at higher risk of having a nonsusceptible isolate to commonly prescribed antibiotics, thereby facilitating improved empiric prescribing practices in uUTI and mitigating development of AMR. Recently, predictive modeling has been used to identify important factors that contribute to risk of nonsusceptibility to antibiotic treatments for uUTI [14, 15]. To date, none have developed a corresponding risk categorization framework that contextualizes different AMR risk profiles.

To address this gap, this study developed and validated predictive models to estimate probabilities of *E. coli* nonsusceptibility to 4 commonly prescribed classes of antibiotic treatments for uUTI, identified key predictors of nonsusceptibility to the 4 classes of antibiotic treatments, and constructed a novel risk categorization framework for nonsusceptibility to the 4 classes of antibiotic treatments.

## METHODS

### Data Source and Study Design

All analyses were conducted using retrospective data from Optum's de-identified Electronic Health Record (EHR) dataset from 1 October 2015 to 29 February 2020. Data were de-identified and compliant with the Health Insurance Portability and Accountability Act; therefore, no institutional review board reviews were required.

Candidate features were evaluated during the 12-month period prior to the susceptibility test result date and were used to develop predictive models for each antibiotic class. The susceptibility test result date was defined as the latest date of an antibiotic susceptibility test result for a urinary *E. coli* isolate collected within 7 days of a UTI diagnosis (*International Classification of Diseases, Tenth Revision, Clinical Modification* codes N30.0, N30.9, and N39.0) without evidence of complicated UTI. Patients with an earlier susceptibility test result date (1 October 2016–31 December 2018) were assigned to the training cohort for model development, while those with a later susceptibility test result date (1 January 2019–29 February 2020) were assigned to the test cohort for model validation.

### Study Population

The study population consisted of female patients ≥12 years of age with a diagnosis of UTI, without evidence of complicated UTI, and a positive urine culture for *E. coli* within ±7 days of the UTI diagnosis. Patients were required to have documentation of pyuria within ±7 days of their diagnosis, and a confirmed antibiotic susceptibility test result of the urinary *E. coli* isolate for at least 1 agent within the 4 antibiotic classes of interest within ±7 days of their UTI diagnosis. A full description of the criteria is provided in the Supplementary Materials.

### Study Endpoints

Four endpoints were evaluated in separate predictive models: nonsusceptibility to NTF, nonsusceptibility to SXT, nonsusceptibility to β-lactams, and nonsusceptibility to fluoroquinolones.

Nonsusceptibility at the class level was defined by a urinary *E. coli* isolate categorized as “intermediate” or “resistant” to at least 1 of the respective antibiotic agent(s) within each class based on susceptibility test results. A detailed list of agents is shown in Supplementary Materials.

### Identification of Candidate Features

Candidate features evaluated in the predictive models were identified based on a targeted literature search and reviewed by 3 clinical experts (R. K. S., J. H., R. C.). They included demographics, clinical characteristics, and microbiology-related characteristics, and are further detailed in the Supplementary Materials.

### Development and Validation of Predictive Models

All analyses were conducted using SAS (version 9.4, SAS Institute, Cary, North Carolina) and Python (version 3.9). Multiple imputation by chained equations (MICE) was used in the development of the predictive models, and it was assumed that all data were missing at random [16].

**Figure 1.**
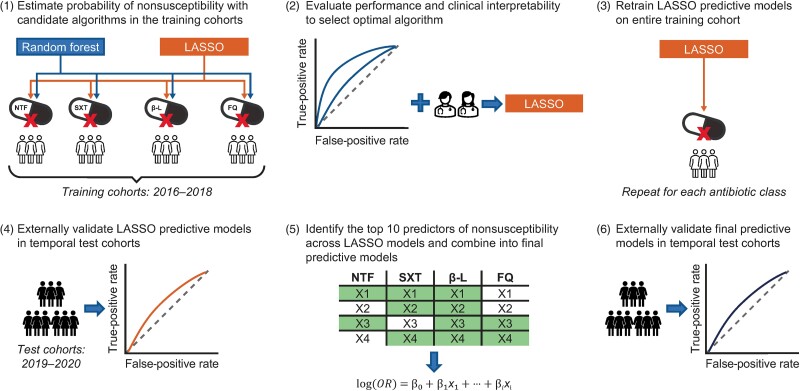
Stepwise analytical approach to developing and validating predictive models to estimate the probability of nonsusceptibility to each of the 4 antibiotic classes. (1) Separate predictive models using least absolute shrinkage and selection operator (LASSO) and random forest algorithms were trained on data from the training cohorts to predict the probability that a urinary *Escherichia coli* isolate of a female patient with uncomplicated urinary tract infection was nonsusceptible to the respective antibiotic class. All candidate features were included in the predictive models and the optimal algorithm was selected via (10,5) stratified nested cross-validation. (2) LASSO was identified as the optimal predictive algorithm across all 4 antibiotic classes based on the mean area under the receiver operating characteristic curve (AUROC) and clinical interpretability. (3) LASSO predictive models were subsequently applied to the entire training cohort for each antibiotic class, and mean AUROC and standard error were derived using internal bootstrap validation. (4) LASSO predictive models were validated using temporal validation in the test cohorts and the AUROC for each model was derived using nonparametric bootstrapping. (5) For each antibiotic class, retained predictors from the LASSO predictive models were ranked according to the magnitude of the absolute value of their standardized log odds ratio (OR). The top 10 predictors with the largest log ORs were identified as key predictors of antibiotic nonsusceptibility to each antibiotic class and combined across the LASSO predictive models to create a comprehensive set of important predictors of nonsusceptibility that were included in 4 logistic regression models of nonsusceptibility to each antibiotic class, considered the final predictive models. (6) The performance of the final predictive models was evaluated using the AUROC and validated using temporal validation in the test cohorts. Abbreviations: β-L, β-lactams; FQ, fluoroquinolones; LASSO, least absolute shrinkage and selection operator; NTF, nitrofurantoin; OR, odds ratio; SXT, trimethoprim-sulfamethoxazole.

Four least absolute shrinkage and selection operator (LASSO) models were developed in the training cohort for each antibiotic class, with the mean area under the receiver operating characteristic curve (AUROC) and standard error (SE) of the LASSO models derived using internal bootstrap validation. The LASSO models were validated in the test cohorts and the SE of the AUROC for each model was derived using 200 bootstrapping samples (Figure 1). Detailed methodology is provided in the Supplementary Materials.

The top 10 predictors with the largest absolute standardized log odds ratio (OR) from each LASSO model were combined into a comprehensive set of predictors given high degree of overlap across the LASSO models. These predictors were included in 4 logistic regression models, which were considered the final predictive models for each of the 4 antibiotic classes. The log OR, OR, 95% confidence interval (CI), and *P* value for each predictor in the 4 final models were reported. The performance of the final models was assessed in the test cohorts based on AUROCs, mean calibration, and calibration plots.

### Development of Clinical Risk Categorization Framework

**Figure 2.**
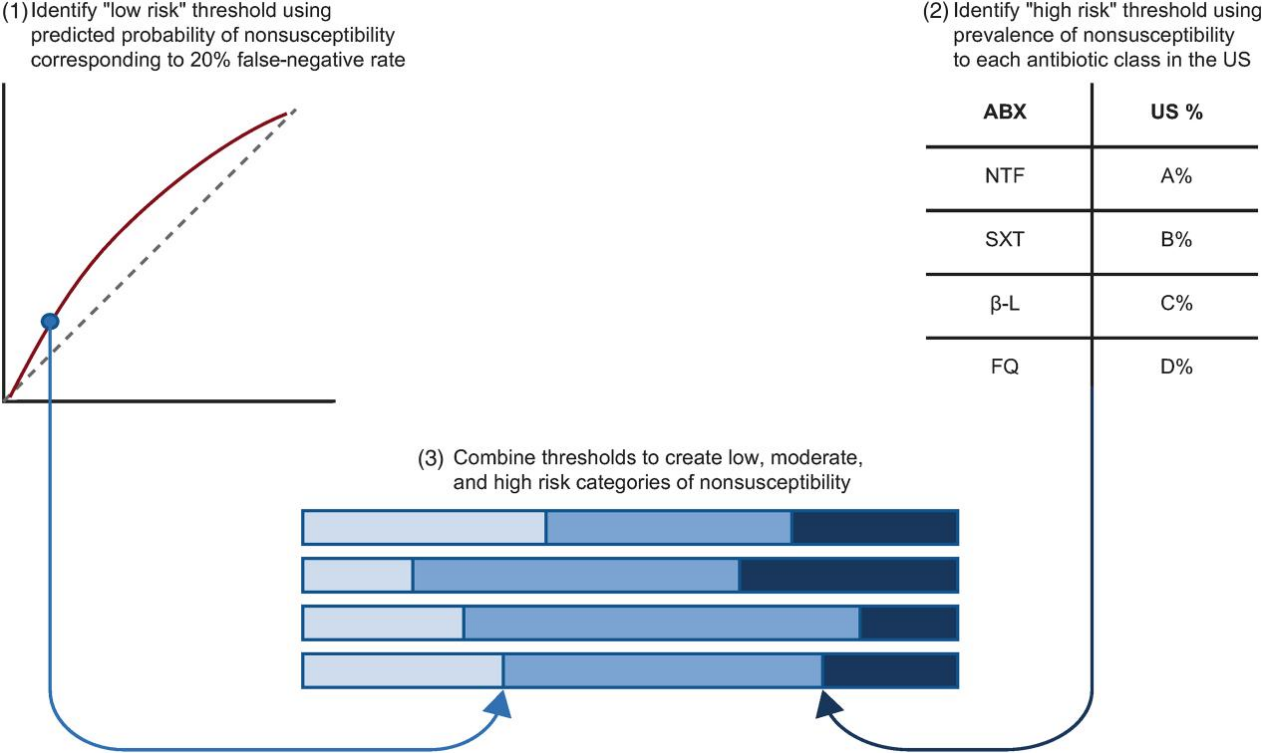
Analytical approach to developing clinical risk categories of nonsusceptibility to each of the 4 antibiotic classes. (1) The predicted probability of nonsusceptibility corresponding to a false-negative rate of 20% for each antibiotic class was used to define the threshold for the “low risk” category to ensure that the maximum proportion of patients with truly nonsusceptible urinary *Escherichia coli* isolates misclassified as having an isolate at “low risk” of nonsusceptibility was 20% for each antibiotic class. (2) The prevalence of nonsusceptibility in the United States (US) for each respective antibiotic class was used to define the threshold for the “high risk” category. As reliable published data on the prevalence of nonsusceptibility to β-lactams in the US were not available, the threshold used to define “high risk” of nonsusceptibility to β-lactams was chosen based on the prevalence of nonsusceptibility observed in the test cohorts of the current study. (3) The thresholds for the “low risk” and “high risk” categories were combined and predicted probabilities of nonsusceptibility falling between these thresholds defined the “moderate risk” category. Abbreviations: ABX, antibiotic; β-L, β-lactams; FQ, fluoroquinolones; NTF, nitrofurantoin; SXT, trimethoprim-sulfamethoxazole; US, United States.

A risk categorization framework was developed based on the predicted probabilities from the final models, published literature on the US prevalence of nonsusceptibility to each antibiotic class [13], and discussions with 3 clinical experts (R. K. S., J. H., R. C.) (Figure 2).

Patients were categorized as having an isolate at “low risk” of nonsusceptibility if their isolate's predicted probability of nonsusceptibility to each antibiotic class was at or below the predicted probability that corresponded to a false-negative rate of 20%, based on clinical input. Patients were categorized as having an isolate at “high risk” of nonsusceptibility if their isolate's predicted probability of nonsusceptibility was higher than the prevalence of nonsusceptibility in the US for each respective antibiotic class (NTF: 3.8%; SXT: 25.4%; fluoroquinolones: 21.1%) [13] or higher than the prevalence of nonsusceptibility observed in the test cohorts when reliable published data were not available (β-lactams: 14.4%). Patients were categorized as having an isolate at “moderate risk” of nonsusceptibility if their isolate's predicted probability of nonsusceptibility fell between the thresholds defining the “low risk” and “high risk” categories (Table 1).

**Table 1.** Prespecified Thresholds Used to Define Clinical Risk Categories for Nonsusceptibility to 4 Antibiotic Classes

Risk Category	Thresholds Used to Define Risk Categories by Antibiotic Class			
	NTF	SXT	β-Lactams	Fluoroquinolones
Low risk	*Predicted probability of nonsusceptibility equal to a false-negative rate of 20%*			
	≤1.9%	≤16.7%	≤8.7%	≤7.7%
Moderate risk	*Predicted probability of nonsusceptibility between low- and high-risk categories*			
	>1.9% and ≤3.8%	>16.7% and ≤25.4%	>8.7% and ≤14.4%	>7.7% and ≤21.1%
High risk	*Predicted probability of nonsusceptibility greater than prevalence of nonsusceptibility in the US corresponding to each antibiotic class*			
	>3.8%	>25.4%	>14.4%^a^	>21.1%

Abbreviations: NTF, nitrofurantoin; SXT, trimethoprim-sulfamethoxazole; US, United States.

^a^As reliable published data on the prevalence of nonsusceptibility to β-lactams in the US were not available, the threshold was based on the prevalence of nonsusceptibility observed in the test cohorts.

## RESULTS

### Study Population

After applying eligibility criteria, 87 487 patients with confirmed antibiotic susceptibility test results to NTF (n = 82 225), SXT (n = 82 921), β-lactams (n = 83 935), and fluoroquinolones (n = 81 223) were included (Supplementary Figure 1). Across the training and test cohorts for each antibiotic class, the mean age of patients ranged from 49.7 to 50.4 years, 85.1%–85.5% were White, and 63.8%–66.3% resided in the Midwest (Supplementary Table 1).

### Identification of Top 10 Predictors of Nonsusceptibility to Each of the 4 Antibiotic Classes

Missing information on candidate features was limited, and results from predictive models were largely consistent using MICE versus a complete case analysis.

Overall, the LASSO models performed well in the test cohorts across all 4 antibiotic classes (Supplementary Table 2). Race and number of previous UTI episodes were top predictive features across all 4 antibiotic classes. US Census Bureau region (particularly residence in the South), prior nonsusceptibility to β-lactams, and prior treatment with fluoroquinolones were top predictive features across 3 of the 4 antibiotic classes (Supplementary Table 3).

### Development and Performance of Final Models

Combining the top 10 predictors from LASSO models for each antibiotic class resulted in a total of 22 features for each logistic regression model. The mean (SE) AUROC of the final models in the test cohorts was 0.67 (0.010) for NTF, 0.66 (0.004) for SXT, 0.66 (0.005) for β-lactams, and 0.72 (0.005) for fluoroquinolones. The mean AUROC of all final models in the test cohorts (Table 2) was like the AUROC from each respective LASSO model (Supplementary Table 2). In the test cohorts, mean (standard deviation) predicted probability of nonsusceptibility was 0.03 (0.04) to NTF, 0.23 (0.17) to SXT, 0.13 (0.11) to β-lactams, and 0.12 (0.16) to fluoroquinolones, and like the observed prevalence of antibiotic nonsusceptibility (Table 2). Calibration plots in the test cohorts suggested stronger agreement between model predictions and observed nonsusceptibility rates for final models developed for nonsusceptibility to β-lactams and fluoroquinolones relative to final models developed for nonsusceptibility to NTF and SXT, likely driven by fewer observations at predicted probabilities >0.50 for the latter models (Supplementary Figure 2).

**Table 2.** Mean Area Under the Receiver Operating Characteristic Curve and Predicted Probabilities of Nonsusceptibility From Final Predictive Models for Antibiotic Treatment Classes

Final Predictive Models Performance Metrics	Antibiotic Treatment Class							
	NTF		SXT		β-Lactams		Fluoroquinolones	
	Training Cohort	Test Cohort	Training Cohort	Test Cohort	Training Cohort	Test Cohort	Training Cohort	Test Cohort
AUROC,^a^ mean (SE)	0.65 (0.008)	0.67 (0.010)	0.66 (0.003)	0.66 (0.004)	0.66 (0.004)	0.66 (0.005)	0.72 (0.004)	0.72 (0.005)
Observed antibiotic nonsusceptibility rates in study population	…	0.033	…	0.239	…	0.144	…	0.131
Predicted probability								
Mean (SD)	0.027 (0.034)	0.029 (0.042)	0.220 (0.153)	0.225 (0.172)	0.119 (0.094)	0.127 (0.110)	0.118 (0.143)	0.123 (0.161)
Median (IQR)	0.021 (0.009)	0.021 (0.009)	0.181 (0.055)	0.180 (0.056)	0.099 (0.041)	0.101 (0.044)	0.086 (0.055)	0.086 (0.055)
Minimum, maximum	0.003, 0.753	0.004, 0.738	0.020, 0.994	0.018, 0.998	0.011, 0.999	0.009, 0.997	0.001, 0.998	0.001, 1.000

Abbreviations: AUROC, area under the receiver operating characteristic curve; IQR, interquartile range; NTF, nitrofurantoin; SD, standard deviation; SE, standard error; SXT, trimethoprim-sulfamethoxazole.

^a^The AUROC was generated by plotting the true-positive rate versus the false-positive rate resulting from different thresholds in the predictive model, and then calculating the area under the curve.

Across the final models for nonsusceptibility to each antibiotic class, prior nonsusceptibility to the respective antibiotic class was associated with significantly higher odds of nonsusceptibility to that antibiotic class (Table 3). Prior nonsusceptibility to a β-lactam agent was a significant predictor of nonsusceptibility to SXT (OR, 1.40 [95% CI: 1.23–1.61]), β-lactams (4.09 [95% CI: 3.56–4.70]), and fluoroquinolones (1.33 [95% CI: 1.15–1.52]; all *P <* .05). Similarly, prior antibiotic treatment with the respective antibiotic class, patient's race, and Census Bureau region were associated with higher odds of nonsusceptibility to the respective antibiotic class across nearly all models. Prior treatment with fluoroquinolones was a significant predictor of nonsusceptibility to SXT (OR, 1.22 [95% CI: 1.10–1.35]), β-lactams (1.43 [95% CI: 1.29–1.59]), and fluoroquinolones (2.54 [95% CI: 2.29–2.82]; all *P <* .05) (Supplementary Table 4).

**Table 3.** Log Odds Ratio and 95% Confidence Intervals of Predictors^a^ of Antibiotic Nonsusceptibility From the Final Predictive Models in the Training Cohorts

Predictors	Logistic Regression Log OR (95% CI)^b^			
	NTF	SXT	β-Lactams	Fluoroquinolones
Demographics^c^				
Age	0.00 (.00–.01)	0.00 (.00–.00)	0.00 (.00–.01)	0.02 (.01–.02)
Race				
White	ref.	ref.	ref.	ref.
Black	0.47 (.28–.65)	0.30 (.22–.38)	0.14 (.03–.25)	0.22 (.11–.33)
Asian	0.09 (−.36 to .53)	0.51 (.35–.67)	0.60 (.42–.79)	0.63 (.44–.81)
Other/unknown	0.21 (−.01 to .42)	0.36 (.28–.45)	0.16 (.05–.27)	0.41 (.30–.52)
Census Bureau region				
Midwest	ref.	ref.	ref.	ref.
West	−0.20 (−.39 to −.01)	−0.09 (−.16 to −.02)	0.04 (−.04 to .13)	−0.10 (−.18 to −.01)
South	0.25 (.08–.42)	0.31 (.24–.38)	0.02 (−.07 to .11)	0.31 (.22–.40)
Northeast	−0.01 (−.22 to .20)	0.16 (.07–.24)	0.11 (.01–.21)	−0.05 (−.15 to .06)
Other/unknown	0.15 (−.10 to .40)	0.16 (.05–.26)	0.05 (−.08 to .18)	0.20 (.06–.33)
Clinical characteristics				
No. of previous UTI episodes (recurrence)^d^	0.12 (.06–.18)	0.10 (.07–.13)	0.10 (.07–.13)	0.14 (.11–.18)
Clinical manifestations of UTI^e^				
Dysuria	−0.15 (−.28 to −.02)	0.04 (−.01 to .09)	−0.18 (−.25 to −.12)	−0.19 (−.25 to −.13)
Urinary frequency	−0.17 (−.30 to −.05)	−0.06 (−.11 to −.01)	−0.17 (−.23 to −.11)	−0.23 (−.29 to −.17)
Antibiotic treatment^f^				
NTF	0.38 (.20–.57)	−0.12 (−.22 to −.02)	0.03 (−.08 to .13)	−0.09 (−.19 to .02)
SXT	−0.01 (−.21 to .19)	0.66 (.57–.75)	0.14 (.04–.24)	0.03 (−.08 to .13)
β-lactams	−0.08 (−.24 to .08)	0.01 (−.06 to .08)	0.26 (.18–.34)	−0.01 (−.09 to .07)
Fluoroquinolones	−0.18 (−.39 to .03)	0.20 (.10–.30)	0.36 (.25–.47)	0.93 (.83–1.04)
Other antibiotics	0.21 (.03–.39)	0.21 (.13–.29)	0.05 (−.05 to .14)	−0.01 (−.11 to .09)
No. of oral antibiotic prescriptions^g^	0.01 (−.02 to .05)	0.02 (.00–.04)	0.04 (.02–.06)	0.04 (.02–.06)
All-cause healthcare resource utilization^h^				
ED visits	0.04 (.01–.08)	0.03 (.01–.05)	0.13 (.10–.16)	0.06 (.02–.09)
Microbiology-related characteristics^h^				
Antibiotic nonsusceptibility				
NTF				
Susceptible	ref.	ref.	ref.	ref.
Nonsusceptible	2.64 (2.41–2.87)	−0.24 (−.45 to −.03)	−0.50 (−.69 to −.32)	0.00 (−.19 to .19)
Not tested/no culture	0.79 (.17–1.40)	−0.20 (−.50 to .10)	−0.39 (−.71 to −.07)	−0.03 (−.35 to .29)
SXT				
Susceptible	ref.	ref.	ref.	ref.
Nonsusceptible	0.72 (.45–.98)	4.67 (4.44–4.90)	−0.09 (−.24 to .06)	−0.32 (−.47 to −.17)
Not tested/no culture	−0.12 (−.82 to .58)	0.94 (.65–1.23)	−0.17 (−.50 to .16)	0.02 (−.31 to .36)
β-lactams				
Susceptible	ref.	ref.	ref.	ref.
Nonsusceptible	−0.27 (−.52 to −.02)	0.34 (.20–.48)	1.41 (1.27–1.55)	0.28 (.14–.42)
Not tested/no culture	−0.71 (−1.56 to .15)	0.43 (.00–.87)	1.88 (1.33–2.43)	−0.05 (−.59 to .50)
Fluoroquinolones				
Susceptible	ref.	ref.	ref.	ref.
Nonsusceptible	0.71 (.43–.99)	0.09 (−.10 to .29)	0.35 (.19–.50)	5.15 (5.00–5.30)
Not tested/no culture	0.36 (−.33 to 1.04)	−0.19 (−.52 to .14)	−0.26 (−.62 to .10)	1.43 (1.06–1.79)
Other antibiotics				
Susceptible	ref.	ref.	ref.	ref.
Nonsusceptible	−0.33 (−.62 to −.05)	0.35 (.16–.54)	0.25 (.09–.40)	0.34 (.18–.49)
Not tested/no culture	−0.13 (−1.06 to .80)	−0.27 (−.72 to .18)	−0.22 (−.73 to .29)	−0.57 (−1.08 to −.06)
Resistant microorganisms isolated from patient samples^i^				
ESBL Enterobacterales				
None present/no culture	ref.	ref.	ref.	ref.
Present and ESBL positive	0.14 (−.43 to .71)	−0.11 (−.50 to .28)	3.24 (2.86–3.61)	0.57 (.19–.94)
Present and ESBL negative	−0.23 (−.69 to .23)	−0.21 (−.45 to .03)	0.12 (−.14 to .38)	−0.05 (−.31 to .21)
Present and not tested	−0.11 (−.61 to .39)	−0.19 (−.44 to .07)	−0.24 (−.52 to .05)	−0.01 (−.29 to .27)
Methicillin-resistant *Staphylococcus aureus*				
None present/no culture	ref.	ref.	ref.	ref.
Present and resistant	−0.45 (−1.68 to .79)	0.10 (−.47 to .68)	−0.58 (−1.18 to .02)	−3.19 (−3.79 to −2.59)
Present and not resistant	−0.19 (−1.12 to .74)	0.09 (−.42 to .60)	0.21 (−.31 to .74)	−2.27 (−2.79 to −1.74)
Present and not tested	−0.03 (−8.65 to 8.58)	−0.06 (−3.52 to 3.40)	−0.14 (−5.73 to 5.45)	−0.79 (−6.38 to 4.80)
Vancomycin-resistant *Enterococcus*				
None present/no culture	ref.	ref.	ref.	ref.
Present and resistant	−0.07 (−6.65 to 6.51)	0.02 (−2.69 to 2.72)	−1.02 (−5.98 to 3.94)	−3.69 (−8.65 to 1.27)
Present and not resistant	0.11 (−.48 to .71)	−0.68 (−1.10 to −.26)	−0.04 (−.43 to .35)	−1.54 (−1.93 to −1.16)
Present and not tested	−0.43 (−3.30 to 2.44)	−0.01 (−1.26 to 1.23)	−1.02 (−3.87 to 1.84)	−2.02 (−4.87 to .84)
Other resistant microorganisms				
None present/no culture	ref.	ref.	ref.	ref.
Present and resistant	−0.36 (−1.21 to .49)	−1.66 (−2.30 to −1.02)	−0.34 (−.84 to 0.17)	−1.38 (−1.89 to −.88)
Present and not resistant	−0.89 (−1.97 to .19)	0.18 (−.33 to .69)	0.28 (−.28 to .84)	0.13 (−.43 to .69)

Abbreviations: CI, confidence interval; ED, emergency department; ESBL, extended-spectrum β-lactamase; NTF, nitrofurantoin; OR, odds ratio; SXT, trimethoprim-sulfamethoxazole; UTI, urinary tract infection.

^a^The predictors included in the final predictive models were selected by taking the union of the top 10 predictors identified by the least absolute shrinkage and selection operator model across the 4 antibiotic classes.

^b^Predictors fitted in the logistic regression were not standardized for ease of interpretation.

^c^Predictors were evaluated on the susceptibility test result date or on the date closest to the susceptibility test result date, unless otherwise specified.

^d^Evaluated during the 12-month period prior to the susceptibility test result date, not including the uncomplicated UTI (uUTI) diagnosis date.

^e^Evaluated during the 12-month period prior to the susceptibility test result date, including the uUTI diagnosis date.

^f^Evaluated from the 6 months prior up to the 3 days preceding the uUTI diagnosis date.

^g^Evaluated during the 12-month period prior to the susceptibility test result date, not including prescriptions on or following the uUTI diagnosis date.

^h^Evaluated during the 12-month period prior to the susceptibility test result date, not including the susceptibility test result date.

^i^Resistant microorganisms were identified from any biological sample, including, but not limited to, urine and blood.

### Development of Clinical Risk Categories of Nonsusceptibility

**Figure 3.**
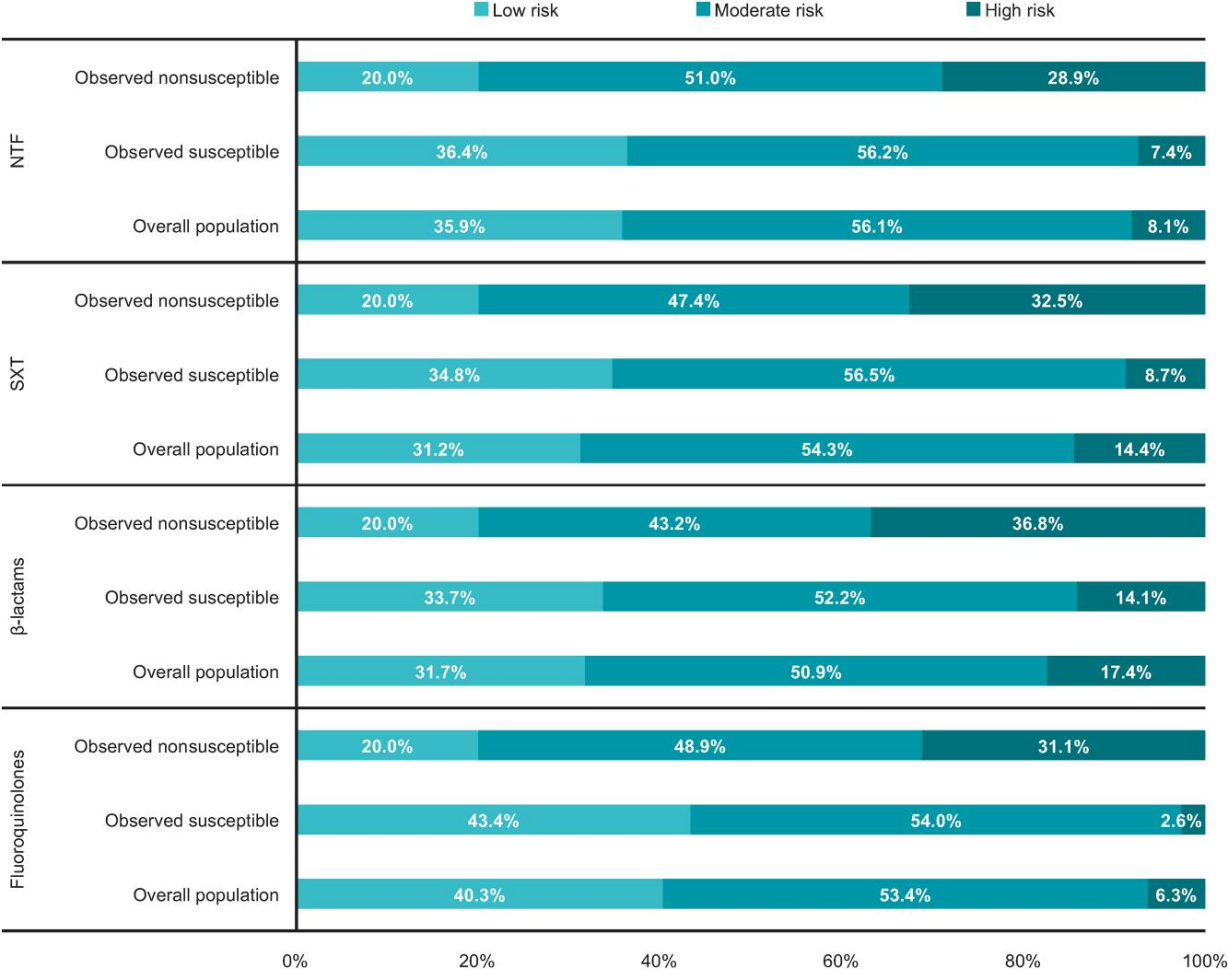
Proportions of patients classified as having *Escherichia coli* isolates at low, moderate, and high risk of nonsusceptibility, overall and by observed susceptibility. Risk categorizations and corresponding results were generated on the test cohort, based on a model fitted to data from the training cohort. Among the overall population, the proportion of patients with isolates in each risk category was defined using the entire test cohort, irrespective of their urinary *E. coli* isolate's observed susceptibility or nonsusceptibility. Observed susceptible refers to patients in the test cohort who did not have a urinary *E. coli* isolate with antibiotic nonsusceptibility to the corresponding antibiotic class. Observed nonsusceptible refers to patients in the test cohort who had a urinary *E. coli* isolate with antibiotic nonsusceptibility to the corresponding antibiotic class. Abbreviations: NTF, nitrofurantoin; SXT, trimethoprim-sulfamethoxazole.

**Figure 4.**
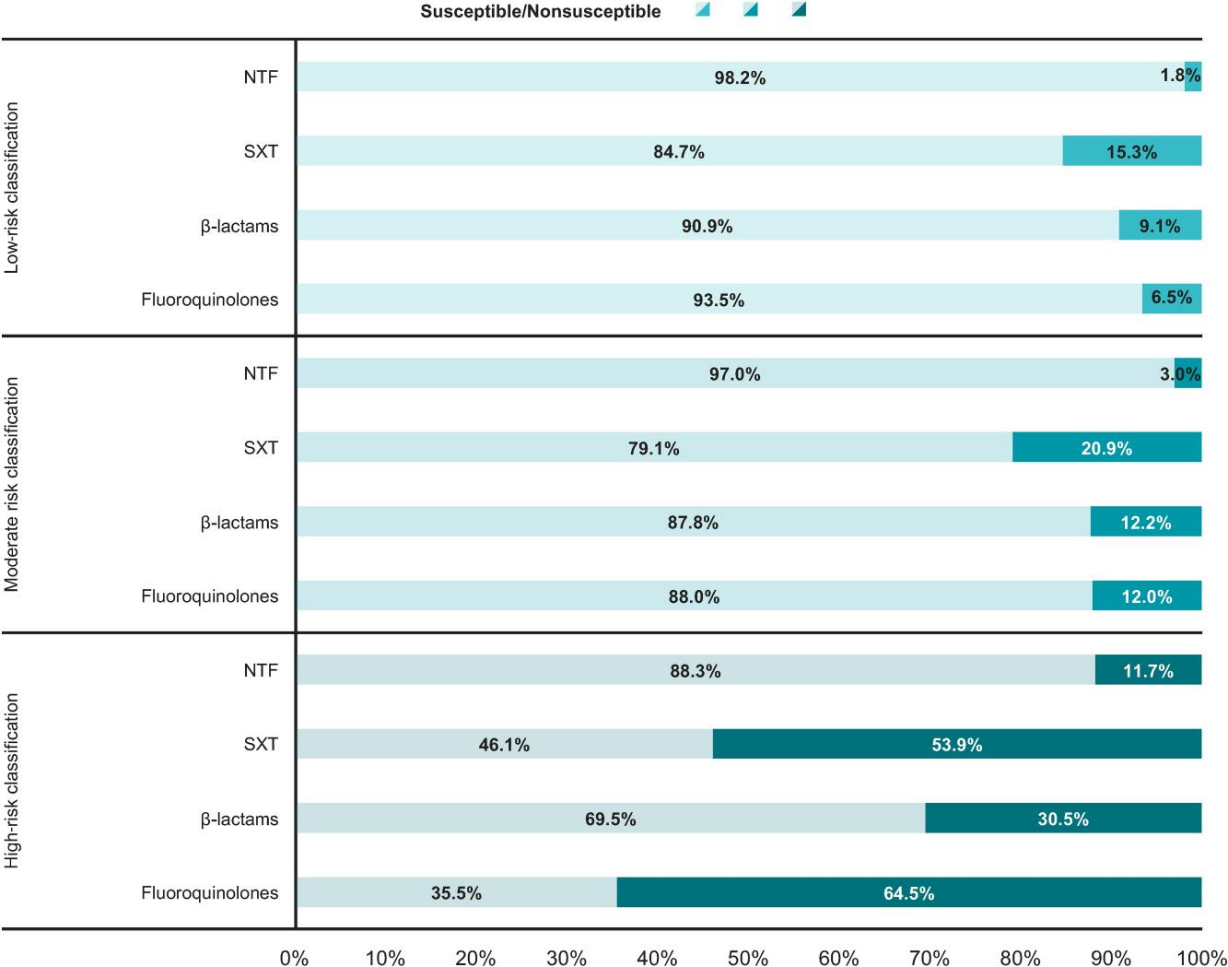
Proportions of patients with urinary *Escherichia coli* isolates observed to be nonsusceptible or susceptible to each of the 4 antibiotic classes stratified by clinical risk categorization. The different shading reflects the proportion of observed susceptible phenotype (lighter shade) versus observed nonsusceptible phenotype (darker shade) to each antibiotic class within each risk category (low, moderate, high). Nonsusceptible refers to patients in the test cohort who had a urinary *E. coli* isolate with antibiotic nonsusceptibility to the corresponding antibiotic class. Susceptible refers to patients in the test cohort who did not have a urinary *E. coli* isolate with antibiotic nonsusceptibility to the corresponding antibiotic class. Risk categorizations and corresponding results were generated on the test cohort, based on a model fitted to data from the training cohort. Abbreviations: NTF, nitrofurantoin; SXT, trimethoprim-sulfamethoxazole.

Using the risk categorization framework, the proportion of patients categorized as having an isolate at high risk of nonsusceptibility was 8.1% for NTF, 14.4% for SXT, 17.4% for β-lactams, and 6.3% for fluoroquinolones. Across all antibiotic classes, the proportion of patients categorized as having an isolate at high risk of nonsusceptibility was 3- to 12-fold higher among patients with truly nonsusceptible isolates than in patients with truly susceptible isolates (Figure 3). Moreover, the proportion of patients with truly nonsusceptible isolates was 3- to 10-fold higher among patients with isolates categorized as high risk of nonsusceptibility versus those with isolates categorized as low risk of nonsusceptibility (Figure 4).

## DISCUSSION

In this study of >87 000 female patients with uUTI, we show how data-driven strategies can mitigate clinical practice challenges in identifying patients with isolates at high risk of nonsusceptibility to commonly prescribed antibiotic treatments. This strategy was achieved through the development of predictive models that elucidate important predictors of antibiotic nonsusceptibility and inform a novel risk categorization framework of *E. coli* nonsusceptibility. To our knowledge, our study is the first to contextualize risk of nonsusceptibility to 4 commonly prescribed antibiotic classes for uUTI using a risk categorization framework informed by predictive modeling. These risk categories may help contextualize the predicted probability of a patient's isolate being nonsusceptible to each of the 4 antibiotic classes. Our findings provide valuable insight to clinicians that help advance the understanding of risk of antibiotic nonsusceptibility in uUTI and inform appropriate empiric prescribing practices.

Using a large EHR database and a comprehensive set of candidate features, we developed predictive models that provide a streamlined approach to quantify a patient's probability of their urinary *E. coli* isolate being nonsusceptible to commonly prescribed antibiotic treatments. Our LASSO predictive models showed modest to good discriminative ability (AUROC, 0.66–0.72) in differentiating between patients with isolates at high risk versus low risk of nonsusceptibility to each antibiotic class. Furthermore, our LASSO models outperform previous predictive models developed for antibiotic nonsusceptibility in uUTI [17]. Although simpler, our final logistic regression models retained the performance observed for the more comprehensive LASSO models, with respect to discrimination and temporal validation, suggesting that the union of the top 10 predictors across the LASSO models were strong predictors of antibiotic nonsusceptibility and that our models will reliably predict antibiotic nonsusceptibility if applied to future data.

Age and race were significant predictors of antibiotic nonsusceptibility in all final models, consistent with existing UTI literature [14, 18]. Our identification of race as a predictor of antibiotic nonsusceptibility may be due in part to its strong correlation with factors (eg, socioeconomic indicators) that were not available in the data and are associated with antibiotic nonsusceptibility [19]. Considering this limitation, the association between race and antibiotic nonsusceptibility observed in the present study should not be endowed with a causal interpretation or used alone to inform treatment decisions. Future studies are warranted to investigate the utility of race as a prognostic factor for antibiotic nonsusceptibility in women with uUTI. Prior nonsusceptibility to β-lactams was a significant predictor of nonsusceptibility to SXT, β-lactams, and fluoroquinolones, suggesting that patients with a previous nonsusceptible isolate to an antibiotic class, in this case β-lactams, may have risk factors that increase future risk of an isolate's nonsusceptibility to other antibiotic classes [20]. In our final models, prior treatment with fluoroquinolones was a significant predictor of nonsusceptibility to all antibiotic classes except NTF, suggesting that nonsusceptibility to NTF may have a differential risk profile with respect to prior antibiotic treatments compared with other antibiotic classes and warrants further investigation.

Our risk categorization framework provides strong separation of risk between truly nonsusceptible versus susceptible isolates and relative to empiric treatment informed by the US prevalence of nonsusceptibility. On average, a random patient would have a 25.4% probability of having a truly nonsusceptible isolate to SXT based on the prevalence of nonsusceptibility to SXT in the US [13]. However, using our risk categorization framework, a patient's probability of having a truly nonsusceptible isolate to SXT increases to 53.9% if their isolate was categorized as high risk of nonsusceptibility, which represents the proportion of patients with truly nonsusceptible isolates categorized as high risk using our framework. Similarly, a patient's probability of having a truly nonsusceptible isolate to SXT will decrease to 15.3% if their isolate was categorized as low risk of nonsusceptibility. Thus, our risk categorization framework provides additional information on the probability that a patient is infected by nonsusceptible *E. coli* beyond what is observed from relying on prevalence rates of antibiotic nonsusceptibility in the US. Overall, our risk categorization approach offers a useful and simplified framework to contextualize the probability of *E. coli* nonsusceptibility to 4 commonly prescribed antibiotic classes in real-world practice.

The findings from our study should be interpreted considering some limitations. First, our predictive models and risk categorization framework do not serve as a decision algorithm to recommend optimal antibiotic treatment of uUTI. Instead, clinicians can derive an isolate's predicted probabilities of nonsusceptibility to 4 antibiotic classes and generate risk profiles of antibiotic nonsusceptibility that can inform empiric treatment for uUTI. Future studies may use the predictive models and risk categorization framework to develop a data visualization tool that may be integrated into clinical workflows to assist with empiric treatment decisions. Second, we were unable to include population-level features, such as local antibiotic nonsusceptibility rates, in our predictive models due to lack of data availability. While local antibiotic nonsusceptibility rates could not be ascertained from the present data, Census Bureau region was included in the predictive models as a proxy feature and identified as a significant predictor of nonsusceptibility across 3 antibiotic classes. Third, our predictive models were developed among patients who were mostly White, from the Midwest, and had an *E. coli*–caused uUTI, which may limit generalizability when applied to diverse US populations with uUTI caused by other uropathogens. Nevertheless, our final models included race and Census Bureau region as predictive features, which may improve their external validity and generalizability. Moreover, as *E. coli* accounts for >80% of uropathogens causing uUTI [3], our final models are expected to be applicable to most real-world patients with uUTIs. Last, the predictive models were anchored on the date of the antibiotic susceptibility test result as opposed to the date of the uUTI diagnosis, which is often when clinicians prescribe antibiotic treatment in real-world practice. In our study population, the average time between the uUTI diagnosis and antibiotic susceptibility test result was <8 days. As such, results are expected to be similar if the predictive models were anchored on the uUTI diagnosis.

Key strengths of our study include the first evaluation of nonsusceptibility to 4 commonly prescribed antibiotic classes indicated for treatment of uUTI in the US using a large, rich EHR database. Second, features included in our predictive models were identified based on a comprehensive literature search and input from clinical experts, yielding the identification of clinically relevant predictors of antibiotic nonsusceptibility. Lastly, we used 2 approaches to contextualize and assess antibiotic nonsusceptibility in uUTI, namely, estimating probabilities of nonsusceptibility to each antibiotic class based on predictive models with good performance and temporal validity, and using a risk categorization framework to construct low, moderate, and high risk categories of antibiotic nonsusceptibility for *E. coli* isolates from patients with uUTI.

## CONCLUSIONS

We developed predictive models and constructed a novel risk categorization framework for *E. coli* nonsusceptibility to 4 commonly prescribed antibiotic classes for uUTI. Our predictive models identified number of previous UTI episodes, prior β-lactam nonsusceptibility, prior fluoroquinolone treatment, Census Bureau region, and race as important predictors of nonsusceptibility to at least 3 antibiotic classes evaluated. Findings from our study provide valuable insight on key patient characteristics to consider when assessing risk of antibiotic nonsusceptibility, thereby advancing the understanding of antibiotic nonsusceptibility in uUTI, and potentially informing optimal treatment strategies in this population. Future studies can provide additional insights by validating our models in different clinical settings and in ex-US populations and recalibrating them to include population-level features such as local antibiotic nonsusceptibility rates.

## Supplementary Data

Supplementary materials are available at *Clinical Infectious Diseases* online. Consisting of data provided by the authors to benefit the reader, the posted materials are not copyedited and are the sole responsibility of the authors, so questions or comments should be addressed to the corresponding author.

## Supplementary Material

ciae171_Supplementary_Data

## Notes

***Acknowledgments.*** The authors thank Sara Dejene for her assistance with editorial support on the manuscript. Editorial support, under the guidance of the authors, was provided by Carol Richter, PhD, of Ashfield MedComms, an Inizio company (Glasgow, UK), and was funded by GSK.

***Data availability.*** Data are not publicly available.

***Disclaimer.*** The study sponsor was involved in several aspects of the research, including the study design, the interpretation of data, and the writing of the manuscript.

***Financial support*.** This work was supported by GSK (study OneCDP ID: 214849).
