# Supplementary material for: Development of Predictive Models to Inform a Novel Risk Categorization Framework for Antibiotic Resistance in Escherichia coli–Caused Uncomplicated Urinary Tract Infection
Source: Clin Infect Dis. 2024 Apr 4;79(2):295–304. doi: 10.1093/cid/ciae171 (PMC11327794; doi:10.1093/cid/ciae171)
Supplement: ciae171_Supplementary_Data [file ciae171_supplementary_data.docx]

**Development of Predictive Models to Inform a Novel Risk Categorization Framework for Antibiotic Resistance in *E. coli-*Causing Uncomplicated Urinary Tract Infection**

**Authors**: Ryan K. Shields, PharmD, MS,^1*^ Wendy Y. Cheng, PhD,^2^ Kalé Kponee-Shovein, ScD,^2^ Daniel Indacochea, PhD,^2^ Chi Gao, ScD,^2^ Fernando Kuwer, MSc,^2^ Ashish V. Joshi, PhD,^3^ Fanny S. Mitrani-Gold, MPH,^3^ Patrick Schwab, PhD,^3^ Diogo Ferrinho, PharmD,^3^ Malena Mahendran, MSc,^2^ Lisa Pinheiro, MFin,^2^ Jimmy Royer, PhD,^2^ Madison T. Preib, MPH,^3^ Jennifer Han, MD, MSCE,^3^ Richard Colgan, MD^4^

**Affiliations:** ^1^Department of Medicine, University of Pittsburgh, Pittsburgh, Pennsylvania, USA; ^2^Analysis Group, Inc., Boston, Massachusetts, USA; ^3^GSK, Collegeville, Pennsylvania, USA; ^4^University of Maryland School of Medicine, Baltimore, Maryland, USA

# Supplementary Materials

Supplementary Section 1. Description of Inclusion and Exclusion Criteria for Study Population

*Inclusion criteria*

- Female
- Documented diagnosis of urinary tract infection (UTI) indicated by the presence of *International Classification of Diseases, Tenth Edition, Clinical Modification* (ICD-10-CM) codes N30.0, N30.9, and N39.0, with a positive urine culture for *Escherichia coli* (*E. coli*) within ±7 days of the diagnosis
- Pyuria within ±7 days of a diagnosis of UTI
  - Pyuria was identified based on having at least one of 1) an ICD-10-CM diagnosis codes (ICD-10-CM: R82.81), 2) a positive leukocyte esterase test, 3) urine white blood cell counts >5 per high-powered field, and 4) the presence of "pyuria" in the natural language processing (NLP) dataset from Optum’s de-identified Electronic Health Record (EHR) dataset
- Treated with one of the four antibiotic classes of interest (ie, NTF, SXT, β-lactams, or fluoroquinolones) within ±5 days of the diagnosis of UTI, to ensure patients were diagnosed with UTI as opposed to asymptomatic bacteriuria
- Confirmed antibiotic susceptibility test result of the urinary *E. coli* isolate for at least one agent within the four antibiotic classes of interest within ±7 days of the UTI diagnosis
- ≥12 years of age on the date of the UTI diagnosis
- ≥12 months of electronic health record (EHR) activity prior to the susceptibility test result date
  - EHR activity was identified by the *First Month Active* field of the Patient dataset, which is defined by Optum's EHR as the earliest month and year with a recorded healthcare activity event in the dataset

*Exclusion criteria*

- Evidence of a complicated UTI during the 12-month period prior to the susceptibility test result date, unless otherwise specified
  - Pregnant
  - Received relevant urological or nephrological procedures (catheter, surgery) within ±28 days of the UTI diagnosis
  - Received a ureteral stent procedure
  - Immunosuppressed or treated with immuno-suppressive therapy
  - Diagnosed with uncontrolled or complicated diabetes (ie, HbA1c >7.0%)
  - Diagnosed with urological abnormalities
  - Intravenous antibiotics within ±28 days of the UTI diagnosis
- Residence in a nursing or retirement home during the 12-month period prior to the susceptibility test result date, including the susceptibility test result date

Supplementary Section 2. Detailed List of Agents Included in Each Antibiotic Class*

*NTF*

- NTF

*Trimethoprim-sulfamethoxazole (SXT)*

- SXT

*Fluoroquinolones*

- Levofloxacin
- Ciprofloxacin
- Ofloxacin

*β-lactams^†^*

- Amoxicillin/potassium clavulanate
- Cefaclor
- Cefadroxil
- Cefdinir
- Cefditoren pivoxil
- Cefixime
- Cefpodoxime proxetil
- Cefprozil
- Ceftibuten
- Cefuroxime
- Cephalexin
- Cephradine
- Loracarbef

* Fosfomycin use in the US is very scarce due to the lack of automated laboratory susceptibility testing methods. In the present study population (N=87,487), susceptibility testing for fosfomycin was rare, with only 1,779 (2.0%) eligible patients with susceptibility test results for fosfomycin identified. Therefore, non-susceptibility to fosfomycin could not be evaluated as a study endpoint.

^†^ All β-lactam agents were considered within one class based on clinical input and the Infectious Diseases Society of America (IDSA) treatment guidelines, which recommend the class of β-lactams as a third-line treatment for uUTI, irrespective of the specific β-lactam agent used.

Supplementary Section 3. Detailed List of Candidate Features for Predictive Models

Candidate features of antibiotic non-susceptibility evaluated in the predictive models were first identified based on a targeted search of published uUTI literature to identify demographics, clinical characteristics, and microbiology-related characteristics potentially associated with antibiotic non-susceptibility, and subsequently reviewed by three clinical experts (RKS, JH, RC) to confirm the clinical relevance of each candidate feature.

*Demographics*

The following demographics were evaluated on the susceptibility test result date:

- Age
- Race
- US census bureau region

*Clinical characteristics*

The following characteristics were evaluated during the 12-month period prior to the susceptibility test result date, not including the susceptibility test result date, unless otherwise specified:

- Number of previous UTI episodes
  - The beginning of a UTI episode was defined using the date of the earliest diagnosis of UTI (identified using ICD-10-CM diagnosis codes and NLP data) during the 12-month period prior to the susceptibility test result date, excluding the uUTI diagnosis. Diagnoses occurring within 28 days of the initial diagnosis were considered part of the same initial episode. The beginning of subsequent episodes required a UTI diagnosis more than 28 days after the last diagnosis of the previous episode.
- Recurrent UTI
  - A patient was considered to have a recurrent UTI if (1) the patient was diagnosed with a recurrent UTI (identified using NLP data), (2) the patient was diagnosed with a history of UTIs (identified using ICD-10-CM diagnosis codes), or (3) the patient had ≥3 UTI episodes during the 12-month period prior to the susceptibility test result date or ≥2 episodes in the 6 months preceding the susceptibility test result date, not including the uUTI diagnosis (diagnoses were identified using ICD-10-CM diagnosis codes and NLP data).
- Clinical manifestations of UTI diagnoses (identified using ICD-10-CM diagnosis codes and NLP data) during the 12-month period prior to the susceptibility test result date, including the uUTI diagnosis
  - Dysuria
  - Urinary frequency
  - Urinary urgency
  - Lower abdominal pain
  - Suprapubic pain
- Antibiotic allergy
  - Penicillin
  - Sulfonamide
  - Other antibiotics
- Treatment with antibiotics in the past 6 months and up to 3 days prior to the uUTI diagnosis date, including but not limited to those used for UTIs
  - NTF
  - SXT
  - Fosfomycin
  - β-lactams
  - Fluoroquinolones
  - Other antibiotics (ie, any agents received that did not fall under the foregoing antibiotic classes)
- Number of prescriptions of oral antibiotic treatment, not including prescriptions on or following the uUTI diagnosis

*Microbiology-related characteristics*

The following characteristics were evaluated during the 12-month period prior to the susceptibility test result date, not including the susceptibility test result date, unless otherwise noted:

- Antibiotic non-susceptibility
  - A patient’s isolate was considered non-susceptible if any of their past isolates (any culture, not limited to urine) were tested for sensitivity to the antibiotic of interest and the result was "Resistant" or "Intermediate". A patient’s isolate was considered susceptible if any of their past isolates were tested for sensitivity to the antibiotic of interest and the result was "Sensitive". A patient isolate’s susceptibility was considered missing if all isolates tested for sensitivity to the antibiotic of interest did not have any available results. If no isolates were tested for sensitivity to the antibiotic of interest or the patient did not have any prior isolates collected, the patient’s isolate was classified as not tested/no culture. Antibiotic non-susceptibilty was reported for the following antibiotic classes:
    - NTF
    - SXT
    - Fosfomycin
    - β-lactams
    - Fluoroquinolones
    - Other antibiotics (ie, non-susceptibility to any agent received that did not fall under the foregoing antibiotic classes)

Prior antibiotic non-susceptibility tests were considered as a baseline feature and did not include the antibiotic susceptibility test corresponding to the susceptibility test result date.

- Resistant microorganisms
  - Extended spectrum beta-lactamase (ESBL) *Enterobacterales*
    - If the microorganism was found "Resistant" or "Intermediate" to ceftriaxone, cefotaxime, ceftazidime, or cefepime then the patient’s isolate was classified as ESBL Positive. If the microorganism was found "Sensitive" then the patient was classified as ESBL Negative.
  - Methicillin-resistant *Staphylococcus aureus*
    - If the microorganism was found "Resistant" to cefoxitin or oxacillin then the patient’s isolate was classified as Resistant. If the microorganism was found "Intermediate" or "Sensitive" then the patient’s isolate was classified as Not Resistant.
  - Vancomycin-resistant *Enterococci*
    - If the microorganism was found "Resistant" to vancomycin then the patient’s isolate was classified as Resistant. If the microorganism was found "Intermediate" or "Sensitive" then the patient’s isolate was classified as Not Resistant.
  - Other resistant microorganisms
    - If a microorganism other than the ones specified were isolated from a patient's sample and tested, and the microorganism was found "Resistant" or "Intermediate" then the patient’s isolate was classified as Resistant. If all microorganisms isolated for a patient were found "Sensitive", the patient’s isolate was classified as Susceptible.

For all microorganisms that were not tested against the antibiotics of interest, the patient’s isolate was classified as present and not tested. If the patient’s isolate did not have the microorganism of interest or the patient did not have any prior isolates collected, the patient’s isolate was classified as not present/no culture.

*Other clinical characteristics*

The following clinical characteristics were evaluated during the 12-month period prior to the susceptibility test result date, not including the susceptibility test result date:

- Urological and nephrological procedures, up to 28 days prior to the susceptibility test result date and not including urological stent
- Acute or semi-acute infections related to antibiotic use (see **Supplementary Table 5** for complete list of diagnosis codes)
- Diabetes
  - Diabetes with no HbA1c test
    - Patients were classified as Diabetes with no HbA1c test if they received a diabetes diagnosis (identified using ICD-10-CM diagnosis codes and NLP data) but did not have an HbA1c test for confirmation. Any patients with an HbA1c result >7.0% in the 12-month period prior to the susceptibility test result date were excluded from the study sample.
  - Controlled diabetes
    - Patients with controlled diabetes included (1) patients noted as having controlled diabetes in the NLP data, (2) patients with a diagnosis of diabetes (identified using ICD-10-CM diagnosis codes and the NLP data) and 4.0%≤ HbA1c ≤7.0%.
  - Prediabetes
    - Patients with prediabetes consisted of patients not classified as having controlled diabetes and having one of the following: (1) noted as having prediabetes in the NLP data, (2) an HbA1c result indicative of prediabetes (5.7%≤ HbA1c ≤7.0%).
  - No diabetes
    - Patients without diabetes consisted of patients not classified as having diabetes, controlled diabetes, or prediabetes.
- Number of emergency department (ED) visits
- Number of hospitalizations

Supplementary Section 4. Analytical Description of Development and Validation of Predictive Models to Estimate the Probability of Non-Susceptibility to Each of the Four Antibiotic Classes

*Development and selection of the optimal predictive algorithm via stratified nested cross validation*

For each of the four antibiotic classes, separate predictive models using least absolute shrinkage and selection operator (LASSO) and random forest algorithms were trained on data from the training cohorts to predict the probability that a urinary *E. coli* isolate of a female patient with uUTI was non-susceptible to the respective antibiotic class. All candidate features were included in the predictive models and the optimal algorithm was selected via stratified nested cross validation. Using (10,5) stratified nested cross validation, the training cohort for each antibiotic class was split into 10 training folds and test folds (outer cross validation). The training folds were further split into five training sub-folds and validation sub-folds (inner cross validation).

For each antibiotic class, hyperparameters for LASSO and random forest were tuned using 5-fold stratified cross validation on the training folds of the training cohort, and the hyperparameters that yielded the highest mean area under the receiver operating characteristic (AUROC) on the validation sub-folds were selected. Using the selected hyperparameters, models using LASSO or random forest algorithms were trained on the training folds of the training cohort for each antibiotic class and evaluated on the 10 test folds of the training cohort. For each antibiotic class, the AUROC was derived for LASSO and random forest based on their mean value across the 10 test folds. The algorithm with the highest mean AUROC across the 10 test folds was selected as the optimal predictive algorithm to use in subsequent analyses.

*Re-training of optimal predictive algorithms to derive and validate predictive models for each of the four antibiotic classes*

Due to the similar AUROCs between the LASSO and random forest algorithms, LASSO was identified as the optimal algorithm for enhanced clinical interpretability across all four antibiotic classes. The LASSO predictive models for each antibiotic class were subsequently applied to the entire training cohort. The mean AUROC and associated standard error (SE) of the LASSO models in the training cohorts were derived using internal bootstrap validation. The LASSO models were validated in the test cohorts using temporal validation and the AUROC for each model was derived using nonparametric bootstrapping.

**Identification of key predictors of non-susceptibility and development of the final predictive models**

For each antibiotic class, retained predictors from the LASSO models were ranked according to the magnitude of the absolute value of their standardized log odds ratio (OR). The top 10 predictors with the largest log ORs were identified as key predictors of antibiotic non-susceptibility to each antibiotic class and combined across the LASSO models to create a comprehensive set of important predictors of non-susceptibility. The comprehensive set of predictors were included in four logistic regression models of non-susceptibility to each antibiotic class and considered as the final predictive models for each of the four antibiotic classes. For each of the four antibiotic classes, the log OR and 95% confidence interval (CI), OR and 95% CI, and *P*-value for each predictor in the final models were reported.

To evaluate the performance of the final models, the AUROC of the models were evaluated in the training and test cohorts and reported using means and SEs. The distribution of the predicted probabilities of non-susceptibility to each antibiotic class was reported along with the observed prevalence of antibiotic non-susceptibility in the test cohorts.

Supplementary Section 5. Optum EHR Database Description

Optum houses a longitudinal repository of clinical and medical administrative data obtained from all internal EHR systems within each of Optum’s provider partners, such as multi-specialty practices, small group practices, physician offices, hospitals, and large ambulatory care centers across the US, which includes more than 107 million patients. Data elements include, but are not limited to, patient demographics, medications prescribed and administered, microbiology and other laboratory results, vital signs, clinical and inpatient stay administrative data, coded diagnoses and procedures, and detailed information from provider notes, such as specific signs and symptoms of disease.

Supplementary Table 1. Summary Statistics for Features included in Predictive Models Across Antibiotic Classes

|  | NTF | | SXT | | β-lactams | | Fluoroquinolones | |
| --- | --- | --- | --- | --- | --- | --- | --- | --- |
|  | Training cohort | Test  cohort | Training cohort | Test  cohort | Training cohort | Test  cohort | Training cohort | Test  cohort |
|  | (N = 52 718) | (N = 29 507) | (N = 53 221) | (N = 29 700) | (N = 53 967) | (N = 29 968) | (N = 52 809) | (N = 28 414) |
| **Demographics^a^** | | | | | | | | |
| Age, years, mean ± SD [median] | 49.7 ± 20.2 [51] | 50.1 ± 20.3 [52] | 49.8 ± 20.2 [51] | 50.1 ± 20.3 [52] | 49.8 ± 20.2 [51] | 50.1 ± 20.3 [52] | 49.8 ± 20.1 [51] | 50.4 ± 20.3 [52] |
| Race, n (%) |  |  |  |  |  |  |  |  |
| White | 44 878 (85.1) | 25 195 (85.4) | 45 329 (85.2) | 25 379 (85.5) | 45 965 (85.2) | 25 607 (85.4) | 44 998 (85.2) | 24 208 (85.2) |
| Black | 3669 (7.0) | 1937 (6.6) | 3684 (6.9) | 1946 (6.6) | 3743 (6.9) | 1967 (6.6) | 3639 (6.9) | 1898 (6.7) |
| Asian | 817 (1.5) | 403 (1.4) | 823 (1.5) | 405 (1.4) | 834 (1.5) | 408 (1.4) | 818 (1.5) | 377 (1.3) |
| Other/Unknown | 3354 (6.4) | 1972 (6.7) | 3385 (6.4) | 1970 (6.6) | 3425 (6.3) | 1986 (6.6) | 3354 (6.4) | 1931 (6.8) |
| Census bureau region, n (%) |  |  |  |  |  |  |  |  |
| Midwest | 34 952 (66.3) | 19 020 (64.5) | 35 072 (65.9) | 19 190 (64.6) | 35 324 (65.5) | 19 247 (64.2) | 34 944 (66.2) | 18 135 (63.8) |
| West | 6255 (11.9) | 3354 (11.4) | 6565 (12.3) | 3368 (11.3) | 6743 (12.5) | 3362 (11.2) | 6262 (11.9) | 3118 (11.0) |
| South | 5297 (10.0) | 2998 (10.2) | 5339 (10.0) | 3004 (10.1) | 5566 (10.3) | 3154 (10.5) | 5324 (10.1) | 3056 (10.8) |
| Northeast | 3855 (7.3) | 2892 (9.8) | 3878 (7.3) | 2880 (9.7) | 3945 (7.3) | 2937 (9.8) | 3931 (7.4) | 2882 (10.1) |
| Other/Unknown | 2359 (4.5) | 1243 (4.2) | 2367 (4.4) | 1258 (4.2) | 2389 (4.4) | 1268 (4.2) | 2348 (4.4) | 1223 (4.3) |
| **Clinical characteristics^b^** | |  |  |  |  |  |  |  |
| Number of previous UTI episodes,^c^ mean ± SD [median] | 0.6 ± 1.0 [0] | 0.7 ± 1.0 [0] | 0.6 ± 1.0 [0] | 0.7 ± 1.0 [0] | 0.6 ± 1.0 [0] | 0.7 ± 1.0 [0] | 0.6 ± 1.0 [0] | 0.7 ± 1.0 [0] |
| Recurrent UTI,^c^ n (%) | 12 601 (23.9) | 7317 (24.8) | 12 711 (23.9) | 7366 (24.8) | 12 835 (23.8) | 7405 (24.7) | 12 659 (24.0) | 7134 (25.1) |
| Clinical manifestations of UTIs,^d^ n (%) | |  |  |  |  |  |  |  |
| Dysuria | 35 689 (67.7) | 19 798 (67.1) | 35 985 (67.6) | 19 923 (67.1) | 36 455 (67.6) | 20 041 (66.9) | 35 781 (67.8) | 18 950 (66.7) |
| Urinary frequency | 31 666 (60.1) | 17 183 (58.2) | 31 859 (59.9) | 17 317 (58.3) | 32 143 (59.6) | 17 385 (58.0) | 31 760 (60.1) | 16 494 (58.0) |
| Urinary urgency | 23 373 (44.3) | 12 603 (42.7) | 23 479 (44.1) | 12 682 (42.7) | 23 648 (43.8) | 12 725 (42.5) | 23 461 (44.4) | 12 076 (42.5) |
| Suprapubic pain | 1570 (3.0) | 1040 (3.5) | 1582 (3.0) | 1044 (3.5) | 1611 (3.0) | 1047 (3.5) | 1586 (3.0) | 1011 (3.6) |
| Lower abdominal pain | 1227 (2.3) | 663 (2.2) | 1233 (2.3) | 667 (2.2) | 1238 (2.3) | 672 (2.2) | 1215 (2.3) | 648 (2.3) |
| Antibiotic allergy, n (%) | |  |  |  |  |  |  |  |
| Penicillin | 767 (1.5) | 430 (1.5) | 778 (1.5) | 426 (1.4) | 804 (1.5) | 441 (1.5) | 782 (1.5) | 417 (1.5) |
| Sulfonamide | 499 (0.9) | 301 (1.0) | 522 (1.0) | 299 (1.0) | 537 (1.0) | 314 (1.0) | 510 (1.0) | 300 (1.1) |
| Other antibiotics | 487 (0.9) | 306 (1.0) | 490 (0.9) | 305 (1.0) | 511 (0.9) | 326 (1.1) | 489 (0.9) | 303 (1.1) |
| Antibiotic treatment,^e^ n (%) | |  |  |  |  |  |  |  |
| NTF | 4269 (8.1) | 3317 (11.2) | 4299 (8.1) | 3331 (11.2) | 4349 (8.1) | 3356 (11.2) | 4272 (8.1) | 3188 (11.2) |
| SXT | 3616 (6.9) | 2169 (7.4) | 3632 (6.8) | 2184 (7.4) | 3693 (6.8) | 2193 (7.3) | 3618 (6.9) | 2087 (7.3) |
| β-lactams | 7625 (14.5) | 5036 (17.1) | 7702 (14.5) | 5079 (17.1) | 7810 (14.5) | 5109 (17.0) | 7616 (14.4) | 4869 (17.1) |
| Fluoroquinolones | 3253 (6.2) | 1530 (5.2) | 3292 (6.2) | 1542 (5.2) | 3341 (6.2) | 1551 (5.2) | 3273 (6.2) | 1499 (5.3) |
| Fosfomycin | 61 (0.1) | 42 (0.1) | 59 (0.1) | 43 (0.1) | 62 (0.1) | 43 (0.1) | 60 (0.1) | 41 (0.1) |
| Other antibiotics | 4742 (9.0) | 2889 (9.8) | 4772 (9.0) | 2911 (9.8) | 4860 (9.0) | 2940 (9.8) | 4751 (9.0) | 2801 (9.9) |
| Prescriptions of oral antibiotic treatment,^f^ n (%) | | |  |  |  |  |  |  |
| 0 | 28 030 (53.2) | 14 573 (49.4) | 28 303 (53.2) | 14 661 (49.4) | 28 676 (53.1) | 14 844 (49.5) | 28 026 (53.1) | 14 047 (49.4) |
| 1 | 11 319 (21.5) | 6551 (22.2) | 11 444 (21.5) | 6569 (22.1) | 11 594 (21.5) | 6620 (22.1) | 11 370 (21.5) | 6286 (22.1) |
| 2 | 6046 (11.5) | 3636 (12.3) | 6078 (11.4) | 3659 (12.3) | 6185 (11.5) | 3680 (12.3) | 6043 (11.4) | 3500 (12.3) |
| 3+ | 7323 (13.9) | 4747 (16.1) | 7396 (13.9) | 4811 (16.2) | 7512 (13.9) | 4824 (16.1) | 7370 (14.0) | 4581 (16.1) |
| **Microbiology-related characteristics^b^** | |  |  |  |  |  |  |  |
| Antibiotic non-susceptibility, n (%) | |  |  |  |  |  |  |  |
| NTF |  |  |  |  |  |  |  |  |
| Non-susceptible | 994 (1.9) | 721 (2.4) | 1002 (1.9) | 724 (2.4) | 1028 (1.9) | 731 (2.4) | 989 (1.9) | 711 (2.5) |
| Susceptible | 8187 (15.5) | 5734 (19.4) | 8312 (15.6) | 5743 (19.3) | 8726 (16.2) | 5873 (19.6) | 8238 (15.6) | 5554 (19.5) |
| Missing | 262 (0.5) | 162 (0.5) | 413 (0.8) | 163 (0.5) | 570 (1.1) | 256 (0.9) | 325 (0.6) | 193 (0.7) |
| Not tested/no culture | 43 275 (82.1) | 22 890 (77.6) | 43 494 (81.7) | 23 070 (77.7) | 43 643 (80.9) | 23 108 (77.1) | 43 257 (81.9) | 21 956 (77.3) |
| SXT |  |  |  |  |  |  |  |  |
| Non-susceptible | 1928 (3.7) | 1420 (4.8) | 1965 (3.7) | 1427 (4.8) | 2031 (3.8) | 1439 (4.8) | 1934 (3.7) | 1381 (4.9) |
| Susceptible | 7315 (13.9) | 5117 (17.3) | 7472 (14.0) | 5122 (17.2) | 7790 (14.4) | 5225 (17.4) | 7380 (14.0) | 4943 (17.4) |
| Missing | 292 (0.6) | 142 (0.5) | 399 (0.7) | 142 (0.5) | 599 (1.1) | 255 (0.9) | 321 (0.6) | 182 (0.6) |
| Not tested/no culture | 43 183 (81.9) | 22 828 (77.4) | 43 385 (81.5) | 23 009 (77.5) | 43 547 (80.7) | 23 049 (76.9) | 43 174 (81.8) | 21 908 (77.1) |
| β-lactams |  |  |  |  |  |  |  |  |
| Non-susceptible | 4663 (8.8) | 3301 (11.2) | 4740 (8.9) | 3325 (11.2) | 4950 (9.2) | 3395 (11.3) | 4699 (8.9) | 3193 (11.2) |
| Susceptible | 5040 (9.6) | 3486 (11.8) | 5275 (9.9) | 3491 (11.8) | 5664 (10.5) | 3639 (12.1) | 5093 (9.6) | 3415 (12.0) |
| Missing | 85 (0.2) | <5 (0.0) | 84 (0.2) | <5 (0.0) | 109 (0.2) | 14 (0.0) | 101 (0.2) | 6 (0.0) |
| Not tested/no culture | 42 930 (81.4) | 22 716 (77.0) | 43 122 (81.0) | 22 880 (77.0) | 43 244 (80.1) | 22 920 (76.5) | 42 916 (81.3) | 21 800 (76.7) |
| Fluoroquinolones |  |  |  |  |  |  |  |  |
| Non-susceptible | 1391 (2.6) | 999 (3.4) | 1404 (2.6) | 1000 (3.4) | 1464 (2.7) | 1018 (3.4) | 1414 (2.7) | 993 (3.5) |
| Susceptible | 7982 (15.1) | 5485 (18.6) | 8143 (15.3) | 5482 (18.5) | 8546 (15.8) | 5635 (18.8) | 8067 (15.3) | 5393 (19.0) |
| Missing | 192 (0.4) | 86 (0.3) | 301 (0.6) | 97 (0.3) | 437 (0.8) | 153 (0.5) | 199 (0.4) | 109 (0.4) |
| Not tested/no culture | 43 153 (81.9) | 22 937 (77.7) | 43 373 (81.5) | 23 121 (77.8) | 43 520 (80.6) | 23 162 (77.3) | 43 129 (81.7) | 21 919 (77.1) |
| Fosfomycin |  |  |  |  |  |  |  |  |
| Non-susceptible | <5 (0.0) | <5 (0.0) | <5 (0.0) | <5 (0.0) | <5 (0.0) | <5 (0.0) | <5 (0.0) | <5 (0.0) |
| Susceptible | 113 (0.2) | 226 (0.8) | 116 (0.2) | 228 (0.8) | 117 (0.2) | 228 (0.8) | 114 (0.2) | 227 (0.8) |
| Missing | 276 (0.5) | <5 (0.0) | 276 (0.5) | <5 (0.0) | 283 (0.5) | <5 (0.0) | 275 (0.5) | <5 (0.0) |
| Not tested/no culture | 52 325 (99.3) | 29 277 (99.2) | 52 825 (99.3) | 29 466 (99.2) | 53 563 (99.3) | 29 733 (99.2) | 52 416 (99.3) | 28 183 (99.2) |
| Other antibiotics |  |  |  |  |  |  |  |  |
| Non-susceptible | 1726 (3.3) | 1266 (4.3) | 1764 (3.3) | 1278 (4.3) | 1798 (3.3) | 1299 (4.3) | 1751 (3.3) | 1224 (4.3) |
| Susceptible | 7845 (14.9) | 5460 (18.5) | 8104 (15.2) | 5458 (18.4) | 8606 (15.9) | 5632 (18.8) | 7909 (15.0) | 5321 (18.7) |
| Missing | 118 (0.2) | 48 (0.2) | 107 (0.2) | 55 (0.2) | 173 (0.3) | 84 (0.3) | 120 (0.2) | 52 (0.2) |
| Not tested/no culture | 43 029 (81.6) | 22 733 (77.0) | 43 246 (81.3) | 22 909 (77.1) | 43 390 (80.4) | 22 953 (76.6) | 43 029 (81.5) | 21 817 (76.8) |
| Resistant microorganisms isolated from patient samples^g^, n (%) | |  |  |  |  |  |  |  |
| *Enterobacterales* species | |  |  |  |  |  |  |  |
| Present | 9445 (17.9) | 6595 (22.4) | 9781 (18.4) | 6626 (22.3) | 10 399 (19.3) | 6853 (22.9) | 9563 (18.1) | 6418 (22.6) |
| ESBL positive | 464 (0.9) | 372 (1.3) | 464 (0.9) | 383 (1.3) | 497 (0.9) | 400 (1.3) | 470 (0.9) | 370 (1.3) |
| ESBL negative | 7005 (13.3) | 5454 (18.5) | 7258 (13.6) | 5446 (18.3) | 7781 (14.4) | 5625 (18.8) | 6962 (13.2) | 5392 (19.0) |
| ESBL missing | 438 (0.8) | 89 (0.3) | 464 (0.9) | 84 (0.3) | 509 (0.9) | 117 (0.4) | 545 (1.0) | 122 (0.4) |
| ESBL not tested | 1538 (2.9) | 680 (2.3) | 1595 (3.0) | 713 (2.4) | 1612 (3.0) | 711 (2.4) | 1586 (3.0) | 534 (1.9) |
| Not present/no culture | 43 273 (82.1) | 22 912 (77.6) | 43 440 (81.6) | 23 074 (77.7) | 43 568 (80.7) | 23 115 (77.1) | 43 246 (81.9) | 21 996 (77.4) |
| *Staphylococcus aureus* |  |  |  |  |  |  |  |  |
| Present | 258 (0.5) | 136 (0.5) | 255 (0.5) | 140 (0.5) | 260 (0.5) | 140 (0.5) | 247 (0.5) | 132 (0.5) |
| Methicillin-resistant | 101 (0.2) | 59 (0.2) | 96 (0.2) | 61 (0.2) | 99 (0.2) | 63 (0.2) | 96 (0.2) | 58 (0.2) |
| Methicillin-susceptible | 151 (0.3) | 74 (0.3) | 152 (0.3) | 75 (0.3) | 153 (0.3) | 76 (0.3) | 148 (0.3) | 71 (0.2) |
| Missing | <5 (0.0) | <5 (0.0) | 5 (0.0) | <5 (0.0) | 6 (0.0) | 0 (0.0) | <5 (0.0) | 0 (0.0) |
| Not tested | <5 (0.0) | <5 (0.0) | <5 (0.0) | <5 (0.0) | <5 (0.0) | <5 (0.0) | <5 (0.0) | <5 (0.0) |
| Not present/no culture | 52 460 (99.5) | 29 371 (99.5) | 52 966 (99.5) | 29 560 (99.5) | 53 707 (99.5) | 29 828 (99.5) | 52 562 (99.5) | 28 282 (99.5) |
| *Enterococcus* species |  |  |  |  |  |  |  |  |
| Present | 287 (0.5) | 194 (0.7) | 287 (0.5) | 201 (0.7) | 297 (0.6) | 196 (0.7) | 294 (0.6) | 193 (0.7) |
| Vancomycin-resistant | <5 (0.0) | <5 (0.0) | <5 (0.0) | <5 (0.0) | <5 (0.0) | <5 (0.0) | <5 (0.0) | <5 (0.0) |
| Vancomycin-susceptible | 257 (0.5) | 188 (0.6) | 256 (0.5) | 191 (0.6) | 262 (0.5) | 190 (0.6) | 259 (0.5) | 187 (0.7) |
| Missing | 8 (0.0) | <5 (0.0) | 11 (0.0) | <5 (0.0) | 11 (0.0) | <5 (0.0) | 11 (0.0) | <5 (0.0) |
| Not tested | 19 (0.0) | <5 (0.0) | 17 (0.0) | 5 (0.0) | 20 (0.0) | <5 (0.0) | 21 (0.0) | <5 (0.0) |
| Not present/no culture | 52 431 (99.5) | 29 313 (99.3) | 52 934 (99.5) | 29 499 (99.3) | 53 670 (99.4) | 29 772 (99.3) | 52 515 (99.4) | 28 221 (99.3) |
| Other microorganisms | |  |  |  |  |  |  |  |
| Present | 254 (0.5) | 184 (0.6) | 263 (0.5) | 188 (0.6) | 261 (0.5) | 193 (0.6) | 251 (0.5) | 182 (0.6) |
| Resistant | 126 (0.2) | 108 (0.4) | 130 (0.2) | 111 (0.4) | 131 (0.2) | 115 (0.4) | 129 (0.2) | 110 (0.4) |
| Susceptible | 117 (0.2) | 76 (0.3) | 121 (0.2) | 77 (0.3) | 120 (0.2) | 78 (0.3) | 113 (0.2) | 72 (0.3) |
| Missing | 11 (0.0) | 0 (0.0) | 12 (0.0) | 0 (0.0) | 10 (0.0) | 0 (0.0) | 9 (0.0) | 0 (0.0) |
| Not present/no culture | 52 464 (99.5) | 29 323 (99.4) | 52 958 (99.5) | 29 512 (99.4) | 53 706 (99.5) | 29 775 (99.4) | 52 558 (99.5) | 28 232 (99.4) |
| **Other characteristics^b^** | |  |  |  |  |  |  |  |
| Urological and nephrological procedure,^h^ n (%) | 528 (1.0) | 403 (1.4) | 535 (1.0) | 407 (1.4) | 540 (1.0) | 410 (1.4) | 529 (1.0) | 398 (1.4) |
| Comorbidities, n (%) | | |  |  |  |  |  |  |
| Acute or semi-acute infections related to antibiotic use | 18 533 (35.2) | 10 908 (37.0) | 18 670 (35.1) | 10 977 (37.0) | 18 966 (35.1) | 11 066 (36.9) | 18 582 (35.2) | 10 480 (36.9) |
| Diabetes |  |  |  |  |  |  |  |  |
| Diabetes with no HbA1c test | 6979 (13.2) | 3296 (11.2) | 7042 (13.2) | 3317 (11.2) | 7132 (13.2) | 3352 (11.2) | 6989 (13.2) | 3237 (11.4) |
| Controlled diabetes | 2941 (5.6) | 1560 (5.3) | 3006 (5.6) | 1578 (5.3) | 3053 (5.7) | 1598 (5.3) | 2978 (5.6) | 1530 (5.4) |
| Prediabetes | 1893 (3.6) | 1456 (4.9) | 1933 (3.6) | 1462 (4.9) | 1959 (3.6) | 1474 (4.9) | 1909 (3.6) | 1423 (5.0) |
| No diabetes | 40 905 (77.6) | 23 195 (78.6) | 41 240 (77.5) | 23 343 (78.6) | 41 823 (77.5) | 23 544 (78.6) | 40 933 (77.5) | 22 224 (78.2) |
| All-cause healthcare resource utilization, mean ± SD [median] | | | |  |  |  |  |  |
| ED visits | 0.5 ± 1.2 [0] | 0.4 ± 1.1 [0] | 0.5 ± 1.2 [0] | 0.4 ± 1.1 [0] | 0.2 ± 0.7 [0] | 0.2 ± 0.8 [0] | 0.5 ± 1.2 [0] | 0.5 ± 1.1 [0] |
| Hospitalizations | 0.1 ± 0.3 [0] | 0.1 ± 0.3 [0] | 0.1 ± 0.3 [0] | 0.1 ± 0.3 [0] | 0.0 ± 0.2 [0] | 0.0 ± 0.2 [0] | 0.1 ± 0.3 [0] | 0.1 ± 0.3 [0] |

Abbreviations: ED, emergency department; ESBL, extended spectrum beta-lactamase; SD, standard deviation; SXT, trimethoprim-sulfamethoxazole; UTI, urinary tract infection; uUTI, uncomplicated urinary tract infection.

^a^Evaluated on the susceptibility test result date or on the date closest to the susceptibility test result date, unless otherwise specified.

^b^Evaluated during the 12-month period prior to the susceptibility test result date, not including the susceptibility test result date.

^c^Evaluated during the 12-month period prior to the susceptibility test result date, not including the uUTI diagnosis date.

^d^Evaluated during the 12-month period prior to the susceptibility test result date, including the uUTI diagnosis date.

^e^Evaluated from the 6 months prior up to the 3 days preceding the uUTI diagnosis date.

^f^Evaluated during the 12-month period prior to the susceptibility test result date, not including prescriptions on or following the uUTI diagnosis date.

^g^Resistant microorganisms were identified from any biological sample, including, but not limited to, urine and blood.

^h^Evaluated up to 28 days prior to the susceptibility test result date and not including urological stent.

Supplementary Table 2. Performance Metrics of LASSO Predictive Models in the Training and Test Cohorts for Each Antibiotic Classes

|  | AUROC,^a^ mean (SE)^b^ | |
| --- | --- | --- |
|  | Training cohort | Test cohort |
| Antibiotic class |  |  |
| NTF | 0.66 (0.008) | 0.67 (0.010) |
| SXT | 0.66 (0.003) | 0.66 (0.004) |
| β-lactams | 0.66 (0.004) | 0.66 (0.005) |
| Fluoroquinolones | 0.72 (0.004) | 0.72 (0.005) |

Abbreviations: AUROC, area under the receiver operating curve; LASSO, least absolute shrinkage and selection operator; NTF, nitrofurantoin; SE, standard error; SXT, trimethoprim-sulfamethoxazole.

^a^The AUROC was generated by plotting the true positive rate versus the false positive rate resulting from different thresholds in the predictive model, and then calculating the area under the curve.

^b^Standard errors were calculated by bootstrapping the relevant cohort (ie, training or test) and computing the corresponding metric using the predictive model, whose coefficients were estimated based on the full training cohort.

Supplementary Table 3. Log ORs and 95% CIs of Predictors^a^ of Antibiotic Non-Susceptibility Identified by LASSO Models

| **Predictors** | **Standardized^a^ LASSO Log OR (95% CI)^b^** | | | |
| --- | --- | --- | --- | --- |
|  | **NTF** | **SXT** | **β-lactams** | **Fluoroquinolones** |
| **Intercept** | -3.78 (-3.84–-3.73) | -1.29 (-1.33–-1.29) | -2.09 (-2.15–-2.09) | -2.21 (-2.26–-2.19) |
| **Demographics^c^** |  |  |  |  |
| Age | 0.04 (0.00–0.10) | -0.02 (-0.04–0.00) | 0.06 (0.05–0.10) | 0.29 (0.26–0.33) |
| Race |  |  |  |  |
| White | ref. | ref. | ref. | ref. |
| Black | 0.12 (0.07–0.16) | 0.07 (0.05–0.10) | 0.02 (0.01–0.06) | 0.05 (0.03–0.08) |
| Asian | 0.01 (-0.06–0.06) | 0.06 (0.05–0.09) | 0.06 (0.05–0.09) | 0.07 (0.05–0.10) |
| Other/Unknown | 0.05 (-0.01–0.09) | 0.08 (0.07–0.11) | 0.02 (0.01–0.06) | 0.09 (0.06–0.13) |
| Census bureau region |  |  |  |  |
| Midwest | ref. | ref. | ref. | ref. |
| West | -0.08 (-0.13–-0.02) | -0.02 (-0.05–-0.01) | — | -0.03 (-0.06–0.00) |
| South | 0.07 (0.03–0.13) | 0.08 (0.07–0.11) | — | 0.09 (0.07–0.12) |
| Northeast | 0.00 (-0.05–0.05) | 0.03 (0.02–0.06) | 0.01 (-0.01–0.04) | -0.01 (-0.05–0.01) |
| Other/Unknown | 0.03 (-0.01–0.07) | 0.02 (0.01–0.05) | — | 0.03 (0.01–0.07) |
| **Clinical characteristics^d^** |  |  |  |  |
| Number of previous UTI episodes^e^ | 0.12 (0.05–0.19) | 0.06 (0.04–0.11) | 0.09 (0.08–0.15) | 0.10 (0.07–0.15) |
| Recurrent UTI^e^ | -0.03 (-0.10–0.04) | 0.02 (0.00–0.06) | — | 0.04 (0.01–0.09) |
| Clinical manifestations of UTI^f^ |  |  |  |  |
| Dysuria | -0.07 (-0.12–-0.01) | — | -0.06 (-0.10–-0.04) | -0.08 (-0.12–-0.05) |
| Urinary frequency | -0.07 (-0.14–0.00) | -0.01 (-0.06–0.00) | -0.04 (-0.08–-0.01) | -0.11 (-0.15–-0.08) |
| Urinary urgency | -0.04 (-0.10–0.02) | — | -0.06 (-0.10–-0.03) | -0.01 (-0.05–0.02) |
| Suprapubic pain | 0.01 (-0.03–0.06) | 0.00 (-0.01–0.03) | 0.02 (0.01–0.06) | 0.00 (-0.02–0.03) |
| Lower abdominal pain | -0.02 (-0.07–0.03) | 0.00 (-0.01–0.03) | — | 0.02 (0.00–0.05) |
| Antibiotic allergy |  |  |  |  |
| Penicillin | 0.02 (-0.02–0.06) | — | -0.03 (-0.08–-0.02) | 0.02 (0.00–0.04) |
| Sulfonamide | 0.01 (-0.06–0.05) | 0.00 (-0.04–0.00) | — | — |
| Other antibiotics | 0.03 (-0.01–0.07) | 0.00 (-0.01–0.04) | — | 0.01 (-0.02–0.04) |
| Antibiotic treatment^g^ |  |  |  |  |
| NTF | 0.10 (0.05–0.15) | -0.01 (-0.06–-0.01) | — | -0.02 (-0.05–0.01) |
| SXT | 0.00 (-0.05–0.04) | 0.15 (0.14–0.19) | 0.02 (0.01–0.06) | — |
| β-lactams | -0.02 (-0.08–0.03) | — | 0.08 (0.06–0.11) | — |
| Fluoroquinolones | -0.05 (-0.10–0.00) | 0.04 (0.02–0.07) | 0.08 (0.06–0.11) | 0.22 (0.19–0.25) |
| Fosfomycin | 0.03 (0.00–0.06) | — | — | -0.01 (-0.06–0.01) |
| Other antibiotics | 0.07 (0.02–0.12) | 0.05 (0.04–0.08) | 0.00 (-0.02–0.04) | — |
| Number of oral antibiotic prescriptions^h^ | 0.03 (-0.02–0.08) | 0.03 (0.00–0.07) | 0.07 (0.04–0.11) | 0.07 (0.03–0.11) |
| **Microbiology-related characteristics^d^** |  |  |  |  |
| Antibiotic non-susceptibility |  |  |  |  |
| NTF |  |  |  |  |
| Susceptible | ref. | ref. | ref. | ref. |
| Not susceptible | 0.36 (0.32–0.40) | -0.01 (-0.06–0.00) | -0.05 (-0.09–-0.04) | — |
| Not tested/no culture | 0.31 (0.00–0.48) | — | — | — |
| SXT |  |  |  |  |
| Susceptible | ref. | ref. | ref. | ref. |
| Not susceptible | 0.13 (0.08–0.19) | 0.85 (0.84–0.94) | — | -0.04 (-0.11–-0.01) |
| Not tested/no culture | -0.07 (-0.29–0.10) | 0.25 (0.22–0.41) | — | — |
| β-lactams |  |  |  |  |
| Susceptible | ref. | ref. | ref. | ref. |
| Not susceptible | -0.08 (-0.13–0.00) | 0.06 (0.05–0.13) | 0.36 (0.36–0.45) | 0.07 (0.03–0.13) |
| Not tested/no culture | -0.25 (-0.51–0.00) | 0.03 (0.00–0.26) | 0.25 (0.40–0.79) | — |
| Fluoroquinolones |  |  |  |  |
| Susceptible | ref. | ref. | ref. | ref. |
| Not susceptible | 0.11 (0.06–0.15) | 0.01 (-0.01–0.05) | 0.06 (0.04–0.09) | 0.81 (0.79–0.88) |
| Not tested/no culture | 0.13 (-0.06–0.36) | — | — | 0.31 (0.31–0.65) |
| Fosfomycin |  |  |  |  |
| Susceptible | ref. | ref. | ref. | ref. |
| Not susceptible | -0.05 (-0.08–0.00) | — | — | -0.02 (-0.06–0.01) |
| Not tested/no culture | -0.02 (-0.05–0.01) | — | -0.02 (-0.06–-0.01) | — |
| Other antibiotics |  |  |  |  |
| Susceptible | ref. | ref. | ref. | ref. |
| Not susceptible | -0.06 (-0.11–-0.01) | 0.04 (0.02–0.09) | 0.03 (0.02–0.07) | 0.04 (0.01–0.10) |
| Not tested/no culture | -0.06 (-0.35–0.20) | — | — | — |
| Resistant microorganisms isolated from patient samples^i^ |  |  |  |  |
| Extended spectrum beta-lactamase *Enterobacterales* |  |  |  |  |
| None present/no culture | ref. | ref. | ref. | ref. |
| Present and ESBL positive | 0.00 (-0.04–0.05) | — | 0.28 (0.26–0.33) | 0.06 (0.01–0.09) |
| Present and ESBL negative | -0.09 (-0.19–0.04) | -0.02 (-0.15–0.00) | — | — |
| Present and not tested | -0.02 (-0.09–0.05) | 0.00 (-0.07–0.01) | -0.05 (-0.08–-0.01) | 0.00 (-0.06–0.04) |
| Methicillin-resistant *Staphylococcus aureus* |  |  |  |  |
| None present/no culture | ref. | ref. | ref. | ref. |
| Present and resistant | -0.02 (-0.16–0.02) | — | -0.02 (-0.06–0.00) | -0.12 (-0.17–-0.08) |
| Present and not resistant | -0.01 (-0.06–0.04) | — | — | -0.10 (-0.18–-0.05) |
| Present and not tested | -0.03 (-0.04–0.00) | — | — | -0.01 (-0.04–0.00) |
| Vancomycin-resistant *Enterococcus* |  |  |  |  |
| None present/no culture | ref. | ref. | ref. | ref. |
| Present and resistant | -0.04 (-0.06–0.00) | — | -0.02 (-0.09–-0.01) | -0.03 (-0.08–0.00) |
| Present and not resistant | 0.01 (-0.03–0.04) | -0.01 (-0.08–-0.01) | — | -0.09 (-0.15–-0.05) |
| Present and not tested | -0.12 (-0.15–-0.02) | — | -0.01 (-0.10–0.00) | -0.02 (-0.14–0.01) |
| Other resistant microorganisms |  |  |  |  |
| None present/no culture | ref. | ref. | ref. | ref. |
| Present and resistant | -0.02 (-0.07–0.01) | -0.05 (-0.13–-0.04) | -0.01 (-0.05–0.00) | -0.05 (-0.12–-0.02) |
| Present and not resistant | -0.04 (-0.14–0.00) | — | — | 0.00 (-0.03–0.04) |
| **Other characteristics^d^** |  |  |  |  |
| Urological and nephrological procedure^j^ | 0.01 (-0.03–0.05) | 0.01 (0.00–0.05) | — | 0.02 (0.00–0.05) |
| Comorbidities |  |  |  |  |
| Acute or semi-acute infections related to antibiotic use | -0.05 (-0.10–0.00) | 0.01 (0.00–0.04) | — | -0.03 (-0.08–-0.01) |
| Diabetes |  |  |  |  |
| No diabetes | ref. | ref. | ref. | ref. |
| Diabetes with no HbA1c test | 0.05 (-0.01–0.10) | — | -0.04 (-0.07–-0.02) | 0.01 (0.00–0.05) |
| Controlled diabetes | 0.05 (0.00–0.09) | 0.01 (-0.01–0.03) | 0.01 (-0.01–0.04) | 0.02 (0.00–0.05) |
| Prediabetes | 0.00 (-0.06–0.05) | — | — | -0.02 (-0.05–0.00) |
| All-cause healthcare resource utilization |  |  |  |  |
| ED visits | 0.05 (0.00–0.08) | 0.03 (0.01–0.05) | 0.07 (0.05–0.10) | 0.06 (0.02–0.08) |
| Hospitalizations | -0.01 (-0.07–0.03) | — | 0.05 (0.03–0.08) | 0.01 (-0.01–0.04) |

Abbreviations: CI, confidence interval; ED, emergency department; LASSO, least absolute shrinkage and selection operator; OR, odds ratio; SD, standard deviation; SXT, trimethoprim-sulfamethoxazole; UTI, urinary tract infection; uUTI, uncomplicated urinary tract infection.

^a^Standardization was performed by rescaling the data such that each feature, except the intercept, had a mean of zero and unit variance. Note that as the data was standardized, the estimated coefficients represent the effect of a marginal increase of one standard deviation in the underlying feature.

^b^Bootstrap CIs were calculated by tuning and training logistic LASSO on 200 bootstrap samples.

^c^Predictors were evaluated on the susceptibility test result date or on the date closest to the susceptibility test result date, unless otherwise specified.

^d^Evaluated during the 12-month period prior to the susceptibility test result date, not including the susceptibility test result date.

^e^Evaluated during the 12-month period prior to the susceptibility test result date, not including the uUTI diagnosis date.

^f^Evaluated during the 12-month period prior to the susceptibility test result date, including the uUTI diagnosis date.

^g^Evaluated from the 6 months prior up to the 3 days preceding the uUTI diagnosis date.

^h^Evaluated during the 12-month period prior to the susceptibility test result date, not including prescriptions on or following the uUTI diagnosis date.

^i^Resistant microorganisms were identified from any biological sample, including, but not limited to, urine and blood.

^j^Evaluated up to 28 days prior to the susceptibility test result date and not including urological stent.

**Supplementary Table 4. ORs and 95% CIs of Predictors^a^ of Antibiotic Non-Susceptibility from the Final Predictive Models in the Training Cohorts**

| Predictors | Logistic Regression OR (95% CI)^b^ | | | |
| --- | --- | --- | --- | --- |
|  | NTF | SXT | β-lactams | Fluoroquinolones |
| **Demographics^c^** |  |  |  |  |
| Age | 1.00 (1.00–1.01)* | 1.00 (1.00–1.00)* | 1.00 (1.00–1.01)* | 1.02 (1.01–1.02)* |
| Race |  |  |  |  |
| White | ref. | ref. | ref. | ref. |
| Black | 1.59 (1.32–1.92)* | 1.35 (1.24–1.47)* | 1.15 (1.03–1.28)* | 1.25 (1.12–1.39)* |
| Asian | 1.09 (0.70–1.69) | 1.67 (1.42–1.96)* | 1.83 (1.52–2.20)* | 1.88 (1.56–2.26)* |
| Other/Unknown | 1.23 (0.99–1.53) | 1.44 (1.32–1.57)* | 1.18 (1.05–1.31)* | 1.50 (1.35–1.68)* |
| Census bureau region |  |  |  |  |
| Midwest | ref. | ref. | ref. | ref. |
| West | 0.82 (0.68–0.99)* | 0.91 (0.85–0.98)* | 1.04 (0.96–1.14) | 0.91 (0.83–0.99)* |
| South | 1.28 (1.09–1.52)* | 1.36 (1.27–1.46)* | 1.02 (0.93–1.12) | 1.36 (1.24–1.49)* |
| Northeast | 0.99 (0.80–1.23) | 1.17 (1.07–1.27)* | 1.11 (1.01–1.23)* | 0.96 (0.86–1.06) |
| Other/Unknown | 1.16 (0.90–1.49) | 1.17 (1.05–1.30)* | 1.05 (0.92–1.20) | 1.22 (1.06–1.39)* |
| **Clinical characteristics** |  |  |  |  |
| Number of previous UTI episodes (recurrence)^d^ | 1.13 (1.06–1.20)* | 1.10 (1.07–1.14)* | 1.11 (1.07–1.14)* | 1.16 (1.12–1.19)* |
| Clinical manifestations of UTI^e^ |  |  |  |  |
| Dysuria | 0.86 (0.76–0.98)* | 1.04 (0.99–1.10) | 0.83 (0.78–0.89)* | 0.83 (0.78–0.88)* |
| Urinary frequency | 0.84 (0.74–0.95)* | 0.94 (0.89–0.99)* | 0.84 (0.79–0.90)* | 0.80 (0.75–0.85)* |
| Antibiotic treatment^f^ |  |  |  |  |
| NTF | 1.46 (1.22–1.76)* | 0.88 (0.80–0.98)* | 1.03 (0.93–1.14) | 0.92 (0.83–1.02) |
| SXT | 0.99 (0.81–1.21) | 1.93 (1.77–2.11)* | 1.15 (1.04–1.28)* | 1.03 (0.93–1.14) |
| β-lactams | 0.92 (0.78–1.08) | 1.01 (0.94–1.08) | 1.29 (1.20–1.40)* | 0.99 (0.92–1.07) |
| Fluoroquinolones | 0.84 (0.67–1.04) | 1.22 (1.10–1.35)* | 1.43 (1.29–1.59)* | 2.54 (2.29–2.82)* |
| Other antibiotics | 1.23 (1.03–1.48)* | 1.24 (1.14–1.34)* | 1.05 (0.95–1.16) | 0.99 (0.90–1.09) |
| Number of oral antibiotic prescriptions^g^ | 1.01 (0.98–1.05) | 1.02 (1.00–1.04)* | 1.04 (1.02–1.06)* | 1.04 (1.02–1.06)* |
| All-cause healthcare resource utilization^h^ |  |  |  |  |
| ED visits | 1.05 (1.01–1.08)* | 1.03 (1.01–1.05)* | 1.14 (1.10–1.18)* | 1.06 (1.02–1.09)* |
| **Microbiology-related characteristics^h^** |  |  |  |  |
| Antibiotic non-susceptibility |  |  |  |  |
| NTF |  |  |  |  |
| Susceptible | ref. | ref. | ref. | ref. |
| Not susceptible | 14.05 (11.14–17.72)* | 0.79 (0.64–0.97)* | 0.60 (0.50–0.73)* | 1.00 (0.83–1.20) |
| Not tested/no culture | 2.19 (1.19–4.05)* | 0.82 (0.61–1.10) | 0.68 (0.49–0.93)* | 0.97 (0.70–1.33) |
| SXT |  |  |  |  |
| Susceptible | ref. | ref. | ref. | ref. |
| Not susceptible | 2.05 (1.56–2.67)* | 106.37 (84.53–133.86)* | 0.91 (0.79–1.06) | 0.73 (0.63–0.84)* |
| Not tested/no culture | 0.89 (0.44–1.79) | 2.55 (1.91–3.41)* | 0.84 (0.60–1.17) | 1.02 (0.74–1.43) |
| β-lactams |  |  |  |  |
| Susceptible | ref. | ref. | ref. | ref. |
| Not susceptible | 0.76 (0.59–0.98)* | 1.40 (1.23–1.61)* | 4.09 (3.56–4.70)* | 1.33 (1.15–1.52)* |
| Not tested/no culture | 0.49 (0.21–1.16) | 1.54 (1.00–2.39) | 6.55 (3.79–11.30)* | 0.95 (0.55–1.65) |
| Fluoroquinolones |  |  |  |  |
| Susceptible | ref. | ref. | ref. | ref. |
| Not susceptible | 2.04 (1.54–2.69)* | 1.10 (0.90–1.34) | 1.41 (1.21–1.65)* | 172.57 (148.04–201.18)* |
| Not tested/no culture | 1.43 (0.72–2.84) | 0.83 (0.60–1.15) | 0.77 (0.54–1.11) | 4.16 (2.89–5.98)* |
| Other antibiotics |  |  |  |  |
| Susceptible | ref. | ref. | ref. | ref. |
| Not susceptible | 0.72 (0.54–0.95)* | 1.42 (1.18–1.72)* | 1.28 (1.10–1.50)* | 1.40 (1.20–1.64)* |
| Not tested/no culture | 0.88 (0.35–2.24) | 0.76 (0.49–1.19) | 0.80 (0.48–1.34) | 0.57 (0.34–0.95)* |
| Resistant microorganisms isolated from patient samples^i^ |  |  |  |  |
| Extended spectrum beta-lactamase *Enterobacterales* |  |  |  |  |
| None present/no culture | ref. | ref. | ref. | ref. |
| Present and ESBL positive | 1.15 (0.65–2.03) | 0.90 (0.61–1.33) | 25.44 (17.45–37.09)* | 1.76 (1.21–2.57)* |
| Present and ESBL negative | 0.80 (0.50–1.26) | 0.81 (0.64–1.03) | 1.13 (0.87–1.46) | 0.95 (0.73–1.23) |
| Present and not tested | 0.90 (0.55–1.48) | 0.83 (0.64–1.07) | 0.79 (0.60–1.05) | 0.99 (0.75–1.31) |
| Methicillin-resistant *Staphylococcus aureus* |  |  |  |  |
| None present/no culture | ref. | ref. | ref. | ref. |
| Present and resistant | 0.64 (0.19–2.21) | 1.11 (0.62–1.97) | 0.56 (0.31–1.02) | 0.04 (0.02–0.07)* |
| Present and not resistant | 0.83 (0.33–2.10) | 1.10 (0.66–1.83) | 1.24 (0.73–2.10) | 0.10 (0.06–0.18)* |
| Present and not tested | 0.97 (0.00–5329.01) | 0.94 (0.03–29.90) | 0.87 (0.00–232.03) | 0.45 (0.00–121.45) |
| Vancomycin-resistant *Enterococcus* |  |  |  |  |
| None present/no culture | ref. | ref. | ref. | ref. |
| Present and resistant | 0.93 (0.00–671.85) | 1.02 (0.07–15.18) | 0.36 (0.00–51.18) | 0.03 (0.00–3.57) |
| Present and not resistant | 1.12 (0.62–2.03) | 0.51 (0.33–0.77)* | 0.96 (0.65–1.41) | 0.21 (0.15–0.31)* |
| Present and not tested | 0.65 (0.04–11.44) | 0.99 (0.28–3.41) | 0.36 (0.02–6.27) | 0.13 (0.01–2.31) |
| Other resistant microorganisms |  |  |  |  |
| None present/no culture | ref. | ref. | ref. | ref. |
| Present and resistant | 0.70 (0.30–1.63) | 0.19 (0.10–0.36)* | 0.71 (0.43–1.18) | 0.25 (0.15–0.41)* |
| Present and not resistant | 0.41 (0.14–1.21) | 1.20 (0.72–1.99) | 1.33 (0.76–2.32) | 1.14 (0.65–2.00) |

**P* < 0.05.

Abbreviations: CI, confidence interval; ED, emergency department; NTF, nitrofurantoin; OR, odds ratio; SXT, trimethoprim-sulfamethoxazole; UTI, urinary tract infection; uUTI, uncomplicated urinary tract infection.

^a^The predictors included in the final predictive models were selected by taking the union of the top 10 predictors identified by the LASSO model across the four antibiotic classes.

^b^Predictors fitted in the logistic regression were not standardized for ease of interpretation.

^c^Predictors were evaluated on the susceptibility test result date or on the date closest to the susceptibility test result date, unless otherwise specified.

^d^Evaluated during the 12-month period prior to the susceptibility test result date, not including the uUTI diagnosis date.

^e^Evaluated during the 12-month period prior to the susceptibility test result date, including the uUTI diagnosis date.

^f^Evaluated from the 6 months prior up to the 3 days preceding the uUTI diagnosis date.

^g^Evaluated during the 12-month period prior to the susceptibility test result date, not including prescriptions on or following the uUTI diagnosis date.

^h^Evaluated during the 12-month period prior to the susceptibility test result date, not including the susceptibility test result date.

^i^Resistant microorganisms were identified from any biological sample, including, but not limited to, urine and blood.

| **Supplementary Table 5. ICD-10-CM Diagnosis Codes for Acute and Semi-acute Infections** | |
| --- | --- |
|  |  |
| **Condition** | **ICD-10-CM Diagnosis Codes** |
| Upper respiratory tract infection | J00, J01, J028, J029, J038, J039, J04, J05, J06, J20, J21, J32 |
| Pneumonia | J09X1, J1008, J11-J18 |
| Otitis | H65-H68 |
| Cellulitis and abscess | L02- L08, K122 |
| Organ infection | A02.21, A36.81, A39.0, A39.1, A39.81, A39.53, A39.52, A39.89, A51.41, A27.81, E06.0, E32.1, D73.3-D73.5, D73.89, G00.0-G00.3, G01, G00.8, G00.9, G04.2, G03.9, G05.3, G05.4, G06.0, G06.1, G06.2, I32, I30.9, I30.8 |
| Bone infection | A02.23, A02.24, A39.83, A54.42, A54.49, A54.41, A54.40, H05.02, M00, M01, M86, M906, M462, M46.30 |
| Surgical site infection (SSI) | K68.11, T85.79XA, T82.6XXA, T82.7XXA, T85.730A, T85.731A, T85.732A, T85.733A, T85.734A, T85.735A, T85.738A, T83.590A, T83.591A, T83.592A, T83.592A, T83.598A, T83.61XA, T83.62XA, T83.69XA, T84.50XA, T84.60XA, T84.7XXA, T85.71XA, T85.79XA |
| Skin and soft tissue infection (SSTI) | A20.1, A21.0, A22.0, A36.3, A46, L08.1, A42.2, B47.9,A42.81, A42.82, A42.89, A43.8, A42.9, A43.9, B47.1, A48.0, A48.8, K90.81, A48.52, M60.009, A28.1, A77.0, A77.1, A77.2, A77.3, A77.40, A77.41, A77.49, A77.8, A79.9, A78, A79.0, A79.1, A79.81, A79.89, A79.9, A68.0, A68.1, A68.9, A44.9, A69.20, B60.0, B60.8, B64, A54.42, K68.12, L02.02, L02.03, L02.12, L02.13, L02.221, L02.222, L02.223, L02.224, L02.225, L02.226, L02.229, L02.231, L02.232, L02.233, L02.234, L02.235, L02.236, L02.239, L02.429, L02.439, L02.529, L02.539, L02.33, L02.629, L02.639, L02.821, L02.828, L02.831, L02.838, L02.92, L02.93, L03.019, L03.029, L03.039, L03.049, K12.2, L03.211, L03.212, L03.213, L03.221, L03.222, L03.319, L03.329, L03.119, L03.129, L03.317, L03.811, L03.818, L03.891, L03.898, L03.90, L03.91, L04.9, L01.00, L01.03, L05.01, L05.02, L05.91, L05.92,L08.0, L88, L08.89, L98.0, E83.2, L08.89, O91.019, O91.011, O91.012, O91.013, O91.02, O91.011, O91.012, O91.013,O91.119, O91.111, O91.112, O91.113, O91.12, O91.23, L73.2, M65.00, M67.80, M67.88, M71.00, M71.80, M60.009, M72.6 |
| Septicemia | A02.1, A20.7, A22.7, A39.4, A39.51, A40.9, A41.2, A41.01, A41.02, A41.1, A40.3, A41.4, A41.50, A41.3, A41.51, A41.52, A41.53, A41.59, A41.89, A41.9, A48.3, I76, I33.0, I39, I33.9, I40.0, R78.81 |
| Clinical sepsis | A41, R65.20, R65.21 |
| Diphtheria, whooping cough, streptococcal throat | A36.0, A36.1, A36.89, A36.2, A36.86, A36.82, A36.83, A36.84, A36.89, A36.9, A37.00, A37.10, A37.80, A37.90, J02.0, J03.00 |
| Viral infections | J11.00, J12.9, J10.1, J11.1, J11.2, J11.81, J11.89, J09.X1, J09.X2, J09.X3, J09.X9, J10.08, A08.0, A08.2, A08.11, A08.19, A08.31, A08.32, A08.39, A08.8, A87.0, A87.8, A87.9, A88.0, A87.2, A87.1, B02.1, B00.3, G02 ,A85.0,A85.1 , A85.8,A88.8, A86, A89, B01.11, B00.4, B05.0, B06.01, B10.01, B10.09, A90, A83.0, A83.1, A83.2, A83.3, A83.4, A83.5, A92.30, A92.31, A92.32, A92.39, A93.8, A94, B02.29, B02.39, B02.8, B02.9, B00.0, A60.9, A60.04, B00.2, B00.50, B00.7, B00.89, B00.81, B00.3, B00.1, B00.82,B00.9, B01.2, B01.12,B01.0,B01.81,B01.89, B01.9, B08.3, B09,L44.4, B05.3, B05.81, B05.1,B05.4,B05.89, B05.9, B10.81, B10.82, B10.89, B06.00, B06.81, B06.89, B06.9, B08.20, B08.21, B08.5, B97.11, A74.0, B30.0, B30.2, B30.1, B30.3, B30.8, A74.0,A74.89, B30.9, B27.90, B25.9, B97.0, B97.12, B97.4, J21.0, J12.0 |
| Oral infections | K04.01, K04.02, K04.7, K04.5, K04.6, K05.00, K05.01, K05.10, K05.11, K05.20, K05.211,K05.212, K05.213, K05.219, K05.221, K05.222, K05.223, K05.229, K05.30, K05.311,K05.312,K05.313, K05.319, K05.321, K05.322, K05.323, K05.329, M27.2, K11.3, K12.2 C, K13.0, K14.0 |
| Gastroenteritis | A00.0, A00.1, A00.9, A01.00, A01.1, A01.2, A01.3, A01.4, A02.0, A02.20, A02.29, A02.8, A02.9, A03.0, A03.1, A03.2, A03.3, A03.8, A03.9, A05.0, A05.1, A05.2, A05.8, A05.3, A05.5, A05.4, A05.9, A04.4, A04.0, A04.1, A04.2, A04.3, A04.8, A04.5, A04.6, A04.9, A09, A21.3, A22.2 |
| Female pelvic infection | A51.0, A51.1, A51.2, A51.31, A51.39, A51.49, A51.43, A51.46, A51.45, A51.49, A51.1, A51.41, A51.32, A51.5, A54.00, A54.29, A54.01, A54.22, A54.23, A54.03, A54.24, A54.29, A54.21, A54.31, A54.32, A54.39, A54.33, A54.6, A54.89, A54.81, A54.83, A54.85, A54.86, A57, A55, A58, M02.30, N34.1, A56.19, A56.4, A56.3, A56.00, A56.2, A56.8, A63.8, A64, N34.1, N34.2, N34.3, N70.01, N70.02, N70.03, N70.11, N70.12, N70.13, N70.91, N70.92, N70.93, N73.0, N73.1, N73.2, N73.3, N73.4, N73.9, N71.0, N71.1, N71.9, N72, N76.2, N76.3 , N75.1, N76.4, N76.0, N76.1, N77.1 |
| Abbreviation: ICD-10-CM, International Classification of Diseases, 10th Revision, Clinical Modification | |

Supplementary Figure 1. Patient Disposition


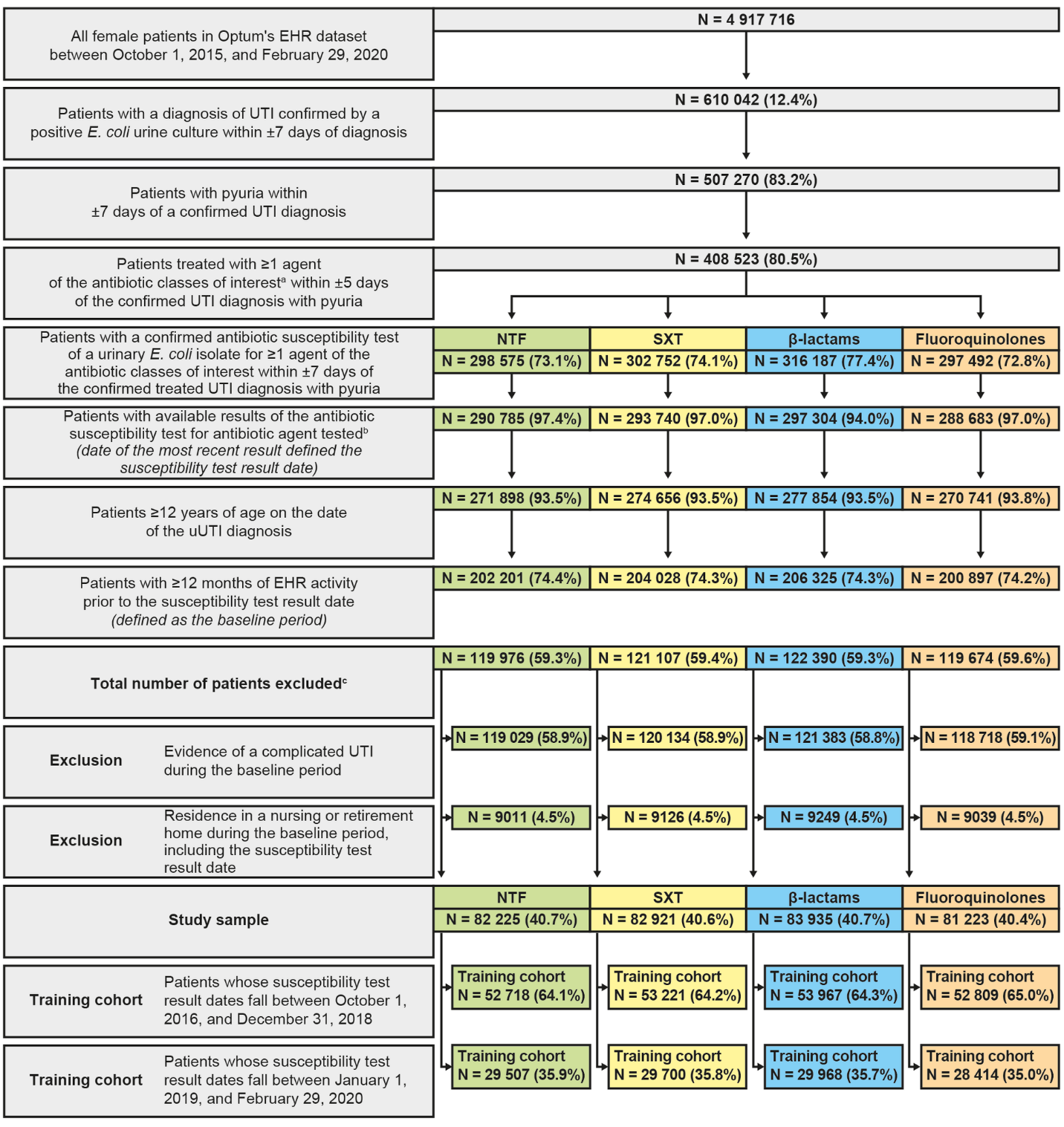


Abbreviations: EHR, electronic health record; NDC, National Drug Code; NTF, nitrofurantoin; SXT, trimethoprim-sulfamethoxazole; UTI, urinary tract infection.

^a^Antibiotic classes of interest included: NTF, SXT, fosfomycin, β-lactams, and fluoroquinolones. Agents of each class were identified using NDCs.

^b^The date of latest antibiotic susceptibility test result, associated with a confirmed, treated UTI with pyuria was defined as the susceptibility test result date. A patient could have up to four different susceptibility test result dates, one for each antibiotic class of interest.

^c^Patients may have met multiple exclusion criterion. Therefore, categories of exclusion criteria are not mutually exclusive and will not sum to the total number of patients excluded.

**Supplementary Figure 2**. **Calibration Plots of Final Predictive Models in the Test Data^a,b^**


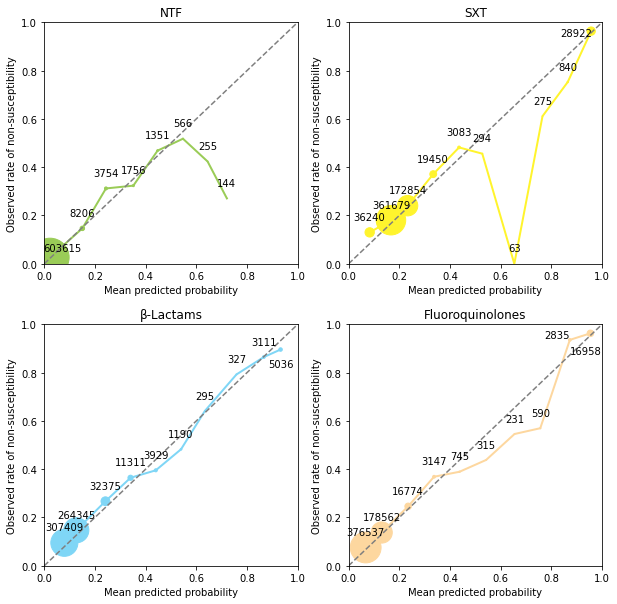


Abbreviations: NTF, nitrofurantoin; SXT, trimethoprim-sulfamethoxazole.

^a^Mean predicted probabilities were derived from each of the four final predictive models for non-susceptibility to NTF, SXT, β-lactams, and fluoroquinolones.

^b^The size of each datapoint represents the relative frequency of predictions and their labels display the respective exact counts.
